# SWI/SNF chromatin remodeling complex is required for initiation of sex-dependent differentiation in mouse germline

**DOI:** 10.1038/s41598-021-03538-8

**Published:** 2021-12-15

**Authors:** Toshiaki Ito, Atsuki Osada, Masami Ohta, Kana Yokota, Akira Nishiyama, Yuichi Niikura, Tomohiko Tamura, Yoichi Sekita, Tohru Kimura

**Affiliations:** 1 Laboratory of Stem Cell Biology, Graduate School of Science, Department of Biosciences, School of Science, Kitasato University, 1-15-1, Kitasato, Minami-ku, Sagamihara, Kanagawa 252-0373 Japan; 2 Department of Immunology, Yokohama City University Graduate School of Medicine, 3-9 Fukuura, Kanazawa-ku, Yokohama, Kanagawa 236-0004 Japan; 3 Faculty of Pharmaceutical Sciences, Josai International University, 1 Gumyo, Togane, Chiba 283-8555 Japan

**Keywords:** Differentiation, Germline development

## Abstract

Sexual reproduction involves the creation of sex-dependent gametes, oocytes and sperm. In mammals, sexually dimorphic differentiation commences in the primordial germ cells (PGCs) in embryonic gonads; PGCs in ovaries and testes differentiate into meiotic primary oocytes and mitotically quiescent prospermatogonia, respectively. Here, we show that the transition from PGCs to sex-specific germ cells was abrogated in conditional knockout mice carrying a null mutation of *Smarcb1* (also known as *Snf5*) gene, which encodes a core subunit of the SWI/SNF chromatin remodeling complex. In female mutant mice, failure to upregulate meiosis-related genes resulted in impaired meiotic entry and progression, including defects in synapsis formation and DNA double strand break repair. Mutant male mice exhibited delayed mitotic arrest and DNA hypomethylation in retrotransposons and imprinted genes, resulting from aberrant expression of genes related to growth and de novo DNA methylation. Collectively, our results demonstrate that the SWI/SNF complex is required for transcriptional reprogramming in the initiation of sex-dependent differentiation of germ cells.

## Introduction

The germline transmits the genomic and epigenomic information to the next generation via the creation of sperms and oocytes. In mice, primordial germ cells (PGCs) arise in the posterior proximal epiblast around embryonic day 7.5 (E7.5)^1,2^. PGCs rapidly proliferate and migrate to the gonads by E11.5. Sex-dependent differentiation starts in gonadal germ cells and is accompanied by dynamic reprogramming of the transcriptome^3–7^. After E13.5, female germ cells enter meiosis and differentiate into primary oocytes, whereas male germ cells enter the G1/G0 phase and differentiate into prospermatogonia. Upon specification from PGCs to oocytes or prospermatogonia, the genes involved in PGC differentiation and pluripotency are downregulated, and sex-dependent transcriptional programs are activated^3^.

A large number of meiosis-related genes are upregulated to initiate and drive meiosis in primary oocytes after E13.5. Retinoic acid (RA) plays a critical role in the induction of meiosis-related genes; RA induces the expression of STRA8 and MEIOSIN, which form a complex and activate meiosis-related genes by binding to the promoters^6,8,9^. On the other hand, recent study has shown that female germ cells enter into meiosis and produce functional oocytes in the absence of all the retinoic acid receptors^10^. In addition, a transcriptional regulator, ZGLP1, which is induced in response to bone morphogenetic protein (BMP), is also a prerequisite for the upregulation of meiosis-related genes^11^. In PGCs, before E12.5, meiosis-related genes are repressed by Polycomb repressive complex 1 (PRC1), which suppresses precocious activation^12^.

In males, RA signaling is inhibited by CYP26B1, which is specifically expressed in the testes and degrades RA^13^. Additionally, an RNA-binding protein, NANOS2, promotes male germ cell differentiation by inhibiting meiotic entry of the prospermatogonia^14–16^. Meanwhile, the genes involved in genome-wide de novo DNA methylation are upregulated in prospermatogonia to accomplish de novo DNA methylation in retrotransposons and imprinted genes^17,18^. However, little is known about the transcriptional mechanisms that initiate and drive sex-dependent differentiation of germ cells.

Switching defective/sucrose nonfermenting (SWI/SNF) complexes are evolutionarily conserved chromatin remodeling complexes that utilize the energy from ATP hydrolysis to mobilize nucleosomes and remodel chromatin^19,20^. SWI/SNF complexes belong to the Trithorax group of complexes that counteract PRC-mediated repression^21^; they are composed of approximately 20 subunits, and mutations in these components are frequently found in human tumors. In tumors, SWI/SNF complexes have been shown to remove PRC1 and PRC2 from promoters with bivalent histone modifications^22–25^.

SWI/SNF complexes also have roles in development. For instance, a lack of SMARCB1 (also known as SNF5, BAF47, or INI1), which is a core component of SWI/SNF complexes, causes peri-implantation lethality in mice^26^. Furthermore, oocyte-specific depletion of *Brg1*, a gene encoding an ATPase subunit of SWI/SNF complexes, leads to aberrant zygotic activation in 2-cell embryos^27^ whereas germ cell-specific deletion of *Brg1* causes meiotic arrest in adult spermatogenesis^28,29^. However, the exact roles of SWI/SNF complexes in embryonic germ cell development are not fully understood. To address this issue, we generated PGC-specific *Snf5* conditional knockout (KO) mice (*Snf5* CKO mice). Our results clearly demonstrate that the SWI/SNF complex is required for the transition from sexually undifferentiated PGCs to female or male germ cells.

## Results

### Generation of PGC-specific *Snf5* CKO mice

To investigate the role of the SWI/SNF complex in embryonic germline development, we used mice carrying a floxed *Snf5* allele and *TNAP-Cre* mice, in which the *Cre* cDNA is knocked into the *Alpl* gene encoding TNAP (tissue nonspecific alkaline phosphatase)^30,31^. The heterozygous *Snf5* KO males (*Snf5*^+*/Δ*^) harboring *TNAP-Cre* were crossed to the homozygous floxed *Snf5* females (*Snf5*^*fx/fx*^) to generate PGC-specific *Snf5* CKO mice (*Snf5*^*fx/Δ*^ + *TNAP-Cre*; Fig. S1A, B). It was reported that Cre-mediated recombination commences in E9.5 PGCs but predominantly takes place in post-migratory phase PGCs from E10.5 to E13.5^31^. RNA-Seq analyses using E15.5 germ cells collected from *Oct4-EGFP* transgenic embryos revealed that the recombination efficiencies at the floxed *Snf5* loci were 90.4% and 80.7% in male and female germlines, respectively (Fig. S1C). Furthermore, we carried out immunohistochemistry using anti-SNF5 antibody in E13.5, E15.5 and E19.5 testes and ovaries (Fig. S1D,E). The percentages of SNF5-positive gem cells were variable among the individual mice. The average percentages of SNF5-positive gem cells in females were 13.7%, 19.1% and 22.7% at E13.5, E15.5 and E19.5, respectively. Those in males were 17.6%, 11.2% and 29.2% at E13.5, E15.5 and E19.5, respectively. Slight increase of SNF5-positive germ cells at E19.5 may reflect the loss of *Snf5*-deficient germ cells by apoptosis, as described below (Fig. S2). In contrast to the germ cells, a majority of gonadal somatic cells in the *Snf5* CKO mice were positive for SNF5.

### Impaired entry into meiosis and mitotic arrest in fetal *Snf5* CKO germ cells

**Figure 1:**
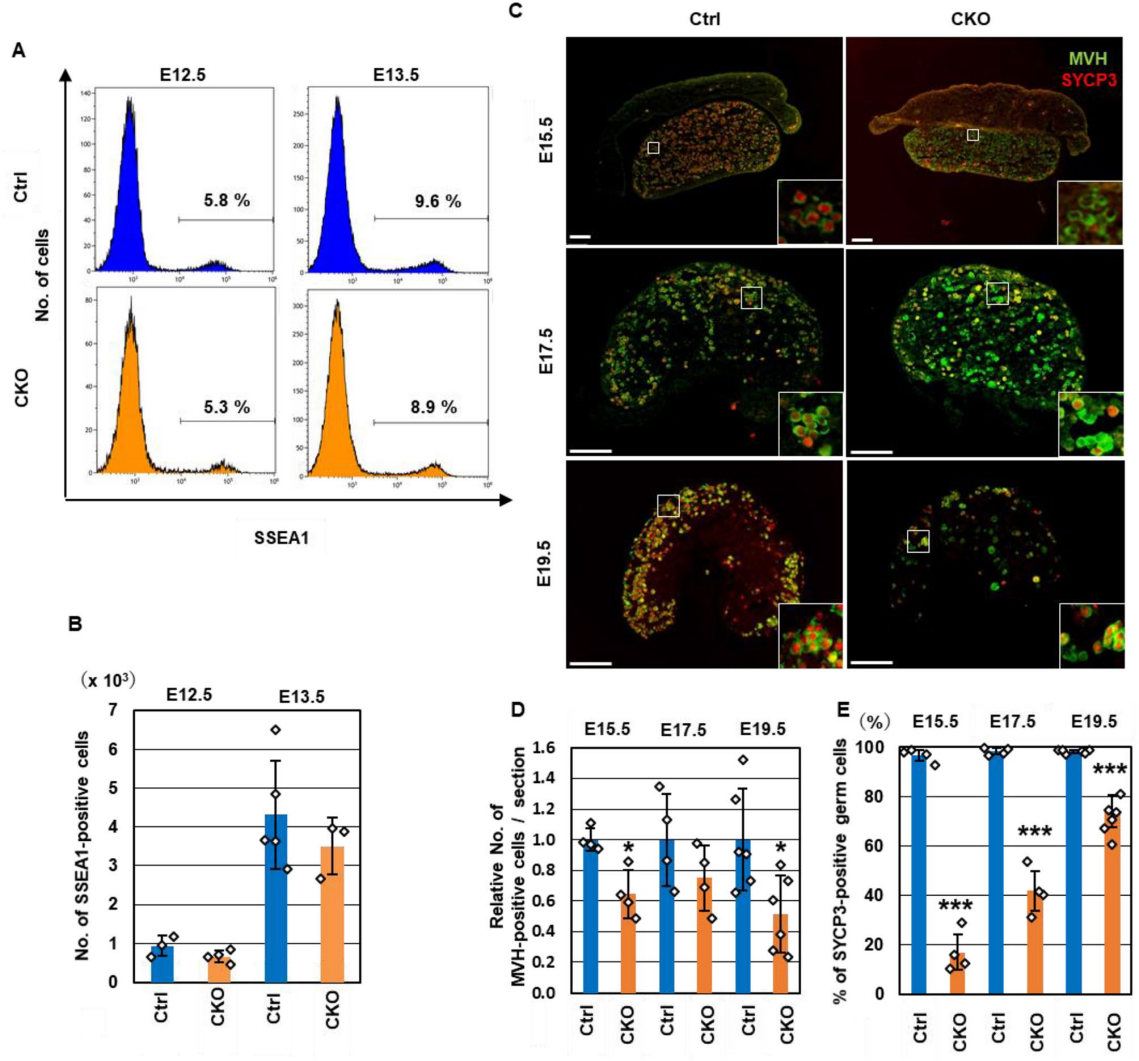
Impaired entry into meiosis in fetal *Snf5* CKO female germ cells. (**A**) Flow cytometry showing the percentages of SSEA1-positive PGCs in female gonads. (**B**) The numbers of PGCs in the E12.5 and E13.5 gonads. The number of SSEA1-positive cells was counted by flow cytometry. The values from individual mice and the mean ± standard deviation are shown. (**C**) Immunohistochemistry of the ovaries in E15.5, E17.5, and E19.5 embryos using antibodies against SYCP3 and MVH. The inserts show higher-magnification views. In the *Snf5* CKO females, SYCP3, a meiosis marker, was not expressed in a number of MVH-positive germ cells. Bars: 100 µm. (**D**) The relative number of MVH-positive germ cells per section. The values from individual mice and the mean ± standard deviation are shown in B, C, and E–G (*P < 0.05, **P < 0.01, ***P < 0.001, Student’s *t*-test). (**E**) The percentages of SYCP3-positive germ cells.

Because Cre-meditated recombination occurs during PGC development in *TNAP-Cre* mice, we first examined fetal ovaries (Fig. 1). The number of SSEA-1-positive PGCs at E12.5 and E13.5 were comparable between the control and *Snf5* CKO females (Fig. 1A,B). Sexually dimorphic differentiation takes place in the germ cells after E13.5; female PGCs enter meiotic prophase and differentiate into primary oocytes. We then investigated whether the female germ cells in *Snf5* CKO mice successfully entered meiosis by immunohistochemistry using an antibody against synaptonemal complex protein 3 (SYCP3), which is expressed from the preleptotene to the diplotene stages of meiotic prophase cells (Fig. 1C–E). In the control females, almost all the mouse vasa homolog (MVH)-positive germ cells expressed SYCP3 from E15.5 to E19.5. In contrast, approximately 20% of the germ cells were positive for SYCP3 at E15.5 in the *Snf5* CKO female mice (Fig. 1C–E). The percentages of SYCP3-positive germ cells in the *Snf5* CKO mice increased gradually until E19.5 but were significantly lower than those in the control mice from E15.5 to E19.5 (Fig. 1E). In addition, there were fewer germ cells in the *Snf5* CKO mice at E15.5 and E19.5 (Fig. 1D). The percentages of cleaved PARP1 (cPARP1)-positive apoptotic gem cells in the *Snf5* CKO mice were comparable to those in the control mice at E15.5, but significantly increased at E19.5 (Fig. S2A,B), indicating the loss of germ cells by apoptosis occurred mainly after perinatal period.

**Figure 2:**
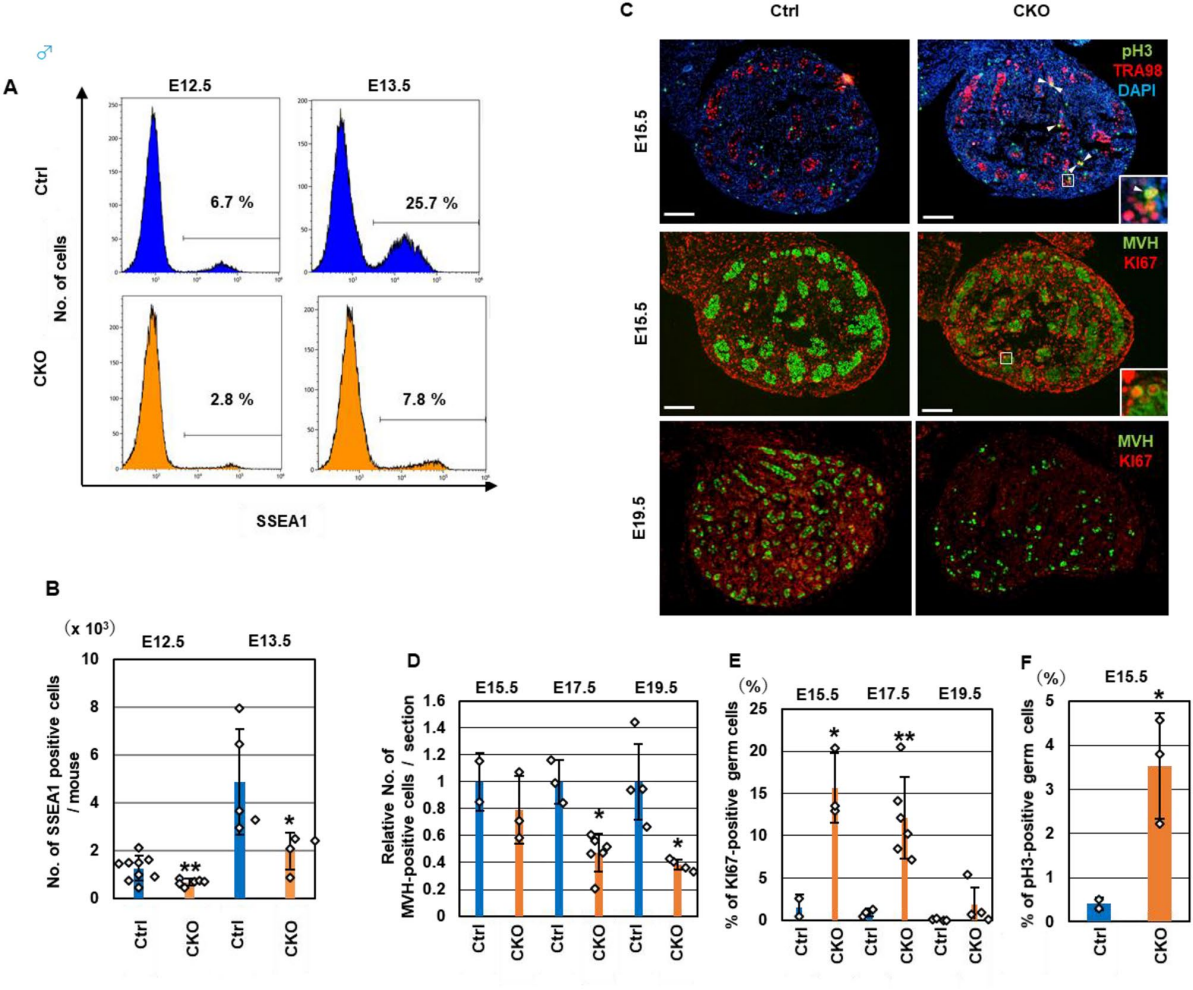
Impaired mitotic arrest in fetal *Snf5* CKO male germ cells. (**A**) Flow cytometry showing the percentages of SSEA1-positive PGCs in male gonads. (**B**) The numbers of PGCs in the E12.5 and E13.5 gonads. The number of SSEA1-positive cells was counted by flow cytometry. The values from individual mice and the mean ± standard deviation are shown. (**C**) Immunohistochemistry of the testes in E15.5 and E19.5 embryos using antibodies against pH3 and MVH (upper panel) or Ki67 and TRA98 (middle and bottom panels). The inserts show higher-magnification views. In *Snf5* CKO males, pH3-positive or Ki67-positive germ cells were detected in the E15.5 testes, but Ki67-positive germ cells were barely detectable in the E19.5 testes. Bars: 100 µm. (**D**) Relative number of MVH-positive germ cells per section. (**E**,**F**) The percentages of Ki67-positive (**E**) or pH3-positive (**F**) germ cells.

Next, we examined the fetal germ cells in the *Snf5* CKO males. The number of PGCs at E12.5 and E13.5 in the *Snf5* CKO males were approximately half of the control males (Fig. 2A,B). We examined whether the germ cells in the *Snf5* CKO males entered mitotic arrest after E15.5 (Fig. 2C–F). Immunohistochemistry using an antibody against phospho-histone H3 (pH3), a marker of M phase cells, showed that pH3-positive germ cells were barely detectable in the testes of the control mice at E15.5, whereas a significantly higher number of pH3-positive germ cells were detected in the *Snf5* CKO mice (Fig. 2C,F). Furthermore, the percentages of germ cells positive for KI67, a marker of proliferation and cell cycling, in the *Snf5* CKO males were significantly higher than those in the control males at E15.5 and E17.5 (Fig. 2C,E). However, KI67-positive germ cells were barely detectable in the *Snf5* CKO males at E19.5, suggesting that male germ cells in the *Snf5* CKO mice eventually entered mitotic arrest. The numbers of germ cells decreased from E15.5 to E19.5 in the *Snf5* CKO males (Fig. 2D). The percentages of apoptotic gem cells in the *Snf5* CKO mice significantly increased at E15.5 (Fig. S2C,D). Transient increase of germ cells in the E15.5 *Snf5* CKO males presumably reflects the prolonged mitotic activity. These results indicate that entry into meiotic prophase and mitotic arrest was impaired in the *Snf5* CKO female and male embryos, respectively.

### Aberrant transcriptional reprogramming in fetal *Snf5* CKO germ cells

**Figure 3:**
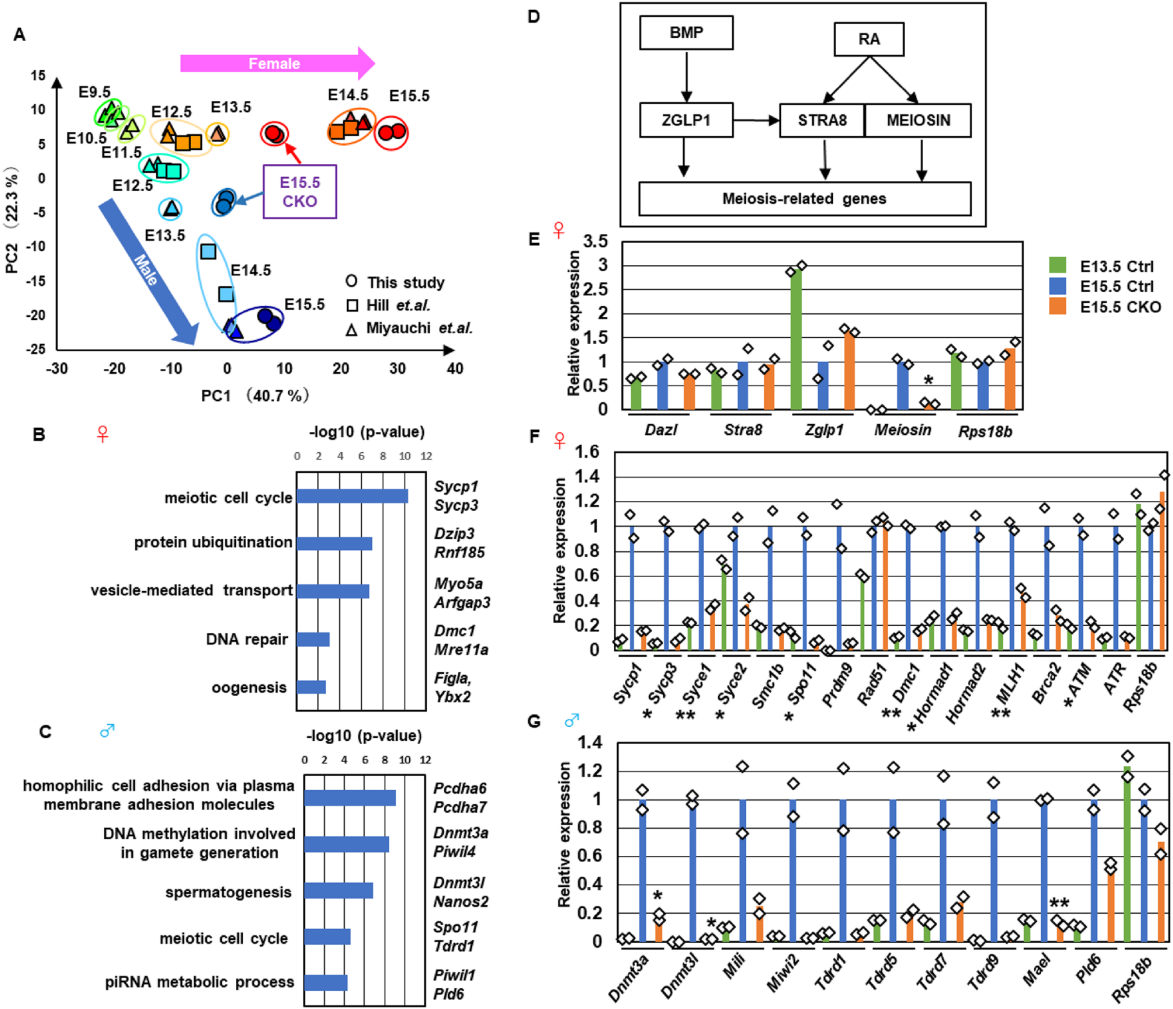
Aberrant transcriptional reprogramming in fetal *Snf5* CKO germ cells. (**A**) PCA of the E15.5 control and *Snf5* CKO germ cells (female, red circles; male, blue circles). The E15.5 germ cells were isolated based on the *Oct4-EGFP* transgene and subjected to RNA-Seq analyses. Data of the E9.5–E15.5 normal germ cells (triangles and squares) have been described previously^4,5^. (**B**,**C**) GO analyses of the genes whose expression levels were significantly lower in female (**B**) and male (**C**) *Snf5* CKO germ cells at E15.5. (**D**) Schema showing the induction of meiosis-related genes in primary oocytes. (**E**) Expression of key genes involved in the induction of meiosis-related genes. The expression levels of control germ cells at E13.5 and E15.5 and the *Snf5* CKO germ cells at E15.5 are shown in (**E**–**G**) (FPKM values of the E15.5 control cells are set as 1.0). The values from biological duplicates and the mean ± standard deviation are shown in (**E**–**G**) (*P < 0.05, **P < 0.01, ***P < 0.001, Student’s *t*-test). (**F**) Expression of representative meiosis-related genes in *Snf5* CKO female germ cells. The expression of ribosomal protein s18b (*Rps18b*) as the internal control is shown in (**F**,**G**). (**G**) Expression of representative de novo DNA methylation-related genes in *Snf5* CKO male germ cells.

To determine the molecular basis of the impaired transition to meiotic prophase and mitotic arrest in the *Snf5* CKO mice, we performed RNA-Seq analyses using the E15.5 female and male germ cells. Transgenic mice expressing enhanced green fluorescent protein (EGFP) under the control of the *Oct4* promoter (*Oct4-EGFP*) were used to purify germ cells^32^. Principal component analyses (PCA) were performed by combining our datasets from E15.5 *Snf5* CKO germ cells with previously reported RNA-Seq datasets from normal germ cells isolated from embryos at E9.5–E15.5 (Fig. 3A)^4,5^. The gene expression patterns of the female and male germ cells progressively diverged from E12.5 to E15.5. The gene expression profiles of the E15.5 *Snf5* CKO germ cells were positioned between the E13.5 and E14.5 germ cells in both females and males (Fig. 3A, Fig. S3B–E). Hierarchical cluster analyses showed that the E15.5 *Snf5* CKO female and male germ cells grouped with the E9.5–E13.5 germ cell cluster (Fig. S3A).

Gene ontology (GO) analysis revealed that key terms related to oogenic fate, such as “meiotic cell cycle”, “DNA repair”, and “oogenesis”, were enriched among the genes whose transcript levels were significantly lower in the *Snf5* CKO females (Fig. 3B). Meanwhile, GO terms related to pluripotency, such as “multicellular organism development” and “stem cell population maintenance”, were enriched among the genes whose transcript levels were significantly higher in the *Snf5* CKO females (Fig. S3F). The genes involved in differentiation and pluripotency in PGCs were not fully downregulated in *Snf5* CKO female germ cells at E15.5 (Fig. S3H).

Two signaling axes play pivotal roles in the upregulation of transcriptional programs necessary for female germ cell differentiation (Fig. 3D). The transcription regulator ZGLP1 is upregulated by BMP in female germ cells after E13.5. Similarly, STRA8 and MEIOSIN, which cooperatively activate the transcription of meiosis-related genes, are upregulated in response to RA. Additionally, an RNA-binding protein, DAZL, is required for sexual differentiation of fetal germ cells in both females and males^33,34^. In the E15.5 *Snf5* CKO germ cells, *Zglp1*, *Stra8*, and *Dazl* were expressed at normal levels, but *Meiosin* was expressed at lower levels than in control mice (Fig. 3E). Despite normal expression of *Zglp1*, *Stra8*, and *Dazl*, a number of the genes involved in synapsis formation, homologous recombination, and DNA repair were expressed at lower levels in the E15.5 *Snf5* CKO germ cells, with the exception of *Rad51*, which encodes a somatic cell-type recombinase (Fig. 3F).

GO terms related to male germ cell differentiation, including “DNA methylation involved in gamete generation”, “spermatogenesis”, and “piRNA metabolic process”, were enriched among the genes whose transcript levels were lower in the *Snf5* CKO males (Fig. 3C). Genome-wide de novo DNA methylation takes place in prospermatogonia. The genes involved in de novo DNA methylation and piRNA-mediated gene silencing were upregulated in the control germ cells from E13.5 to E15.5, but not in the *Snf5* CKO germ cells (Fig. 3G). On the other hand, GO terms such as “cell cycle”, “mitotic nuclear division”, and “G1/S transition of mitotic cell cycle” were enriched among the upregulated genes (Fig. S3G), and PCA of growth-related genes showed that the *Snf5* CKO cells grouped with the E9.5–E13.5 germ cell cluster (Fig. S3J). The aberrant expression of genes related to cell cycle regulation may account for the impaired mitotic arrest in *Snf5* CKO males. Additionally, similar to female germ cells, genes involved in PGC differentiation were not downregulated in *Snf5* CKO male germ cells at E15.5 (Fig. S3I). Collectively, these findings demonstrate that *Snf5* is required for sex-dependent transcriptional reprogramming in both female and male fetal germlines.

### Impaired meiotic progression of *Snf5* CKO female germ cells

**Figure 4:**
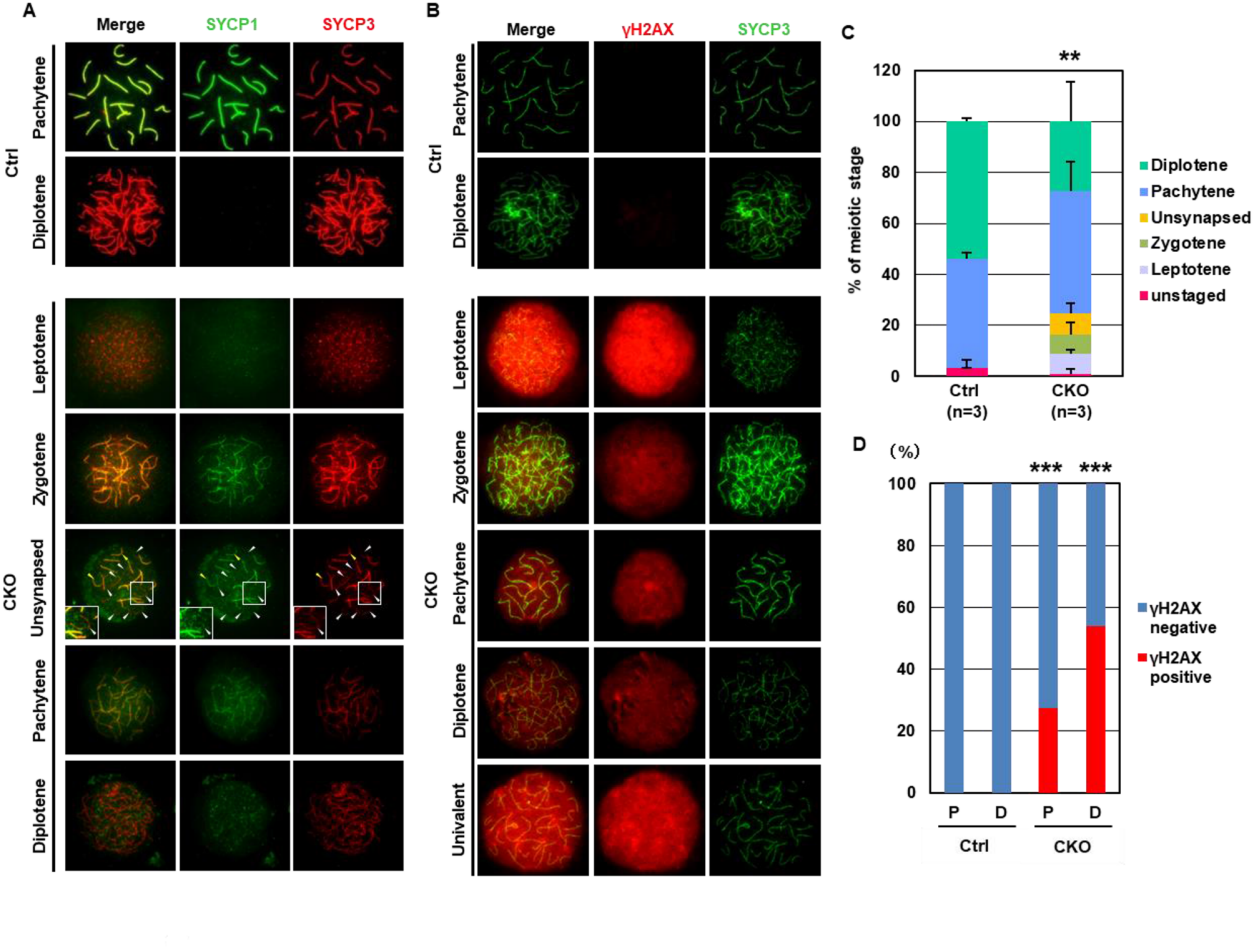
Impaired meiotic progression of *Snf5* CKO female germ cells. (**A**,**B**) The chromosome spreads in E19.5 ovaries immunostained with antibodies against SYCP1 and SYCP3 (**A**), or with antibodies against γH2AX and SYCP3 (**B**). The inserts show higher-magnification views. Yellow and white arrowheads indicate synapsed and unsynapsed chromosomes, respectively. (**C**) Distribution of each stage of meiotic prophase. The distribution was significantly different between the control and *Snf5* CKO females (**P < 0.01, *χ*^2^ test). The mean ± standard deviation is shown. Approximately 30 cells were analyzed for each mouse. (**D**) The percentages of γH2AX-positive pachynema (P) and diplonema (D). The percentages were significantly higher in both the pachynema and diplonema of the *Snf5* CKO females (***P < 0.001, *χ*^2^ test). The number of cells analyzed are as follows: pachynema and diplonema in the control mice, 34 cells from three females and 55 cells from three females; pachynema and diplonema in the *Snf5* CKO mice, 29 cells from three females and 39 cells from three females.

Our results demonstrated that initiation of meiotic prophase was impaired in *Snf5* CKO females, presumably due to the lower expression of meiosis-related genes. On the other hand, histological analyses showed a gradual increase in SYCP3-positive germ cells until E19.5 in the *Snf5* CKO females (Fig. 1C,E). Next, we evaluated the meiotic stages using immunohistochemistry of the chromatin spreads in the E19.5 germ cells using antibodies against SYCP3 and SYCP1, which are components of the axial and central elements of synaptonemal complexes, respectively (Fig. 4A,C). We observed only pachytene and diplotene germ cells in the control females at E19.5. In contrast, in the *Snf5* CKO females, we observed not only pachytene and diplotene cells but also leptotene and zygotene germ cells. In addition, the immunohistochemical signals for SYCP3 and SYCP1 were much weaker in the pachynema and diplonema of the *Snf5* CKO females compared with the control females (Fig. 4A). Furthermore, pachytene-like cells harboring unsynapsed chromosomes were detected in *Snf5* CKO females (Fig. 4A,C).

Genome-wide introduction of double-stranded breaks (DSBs) is a prerequisite for the initiation of meiotic recombination, which is followed by synapsis formation, homologous recombination between homologous chromosomes, and subsequent chiasma formation. GO analysis revealed that genes related to “DNA repair” were enriched among the genes whose expression levels were lower in *Snf5* CKO females (Fig. 3B). We, therefore, examined the chromosome spreads in the E19.5 germ cells using an antibody against γH2AX, a marker of DSBs. In the control mice, no γH2AX-positive germ cells were detected, and this was because all oocytes entered the pachytene and diplotene stages at E19.5. However, approximately 30% of pachynema and 50% of diplonema were γH2AX-positive in the *Snf5* CKO females (Fig. 4B,D). In addition to asynapsis in pachynema (Fig. 4A,C), diplonema carrying univalent chromosomes were detected in the *Snf5* KO females (Fig. 4B), suggesting that asynapsis in pachynema led to defective chiasma formation. These results collectively suggest that synapsis formation, DNA repair, and chiasma formation were compromised in the fetal germ cells of *Snf5* KO females.

### Impaired de novo DNA methylation in *Snf5* CKO male germ cells

**Figure 5:**
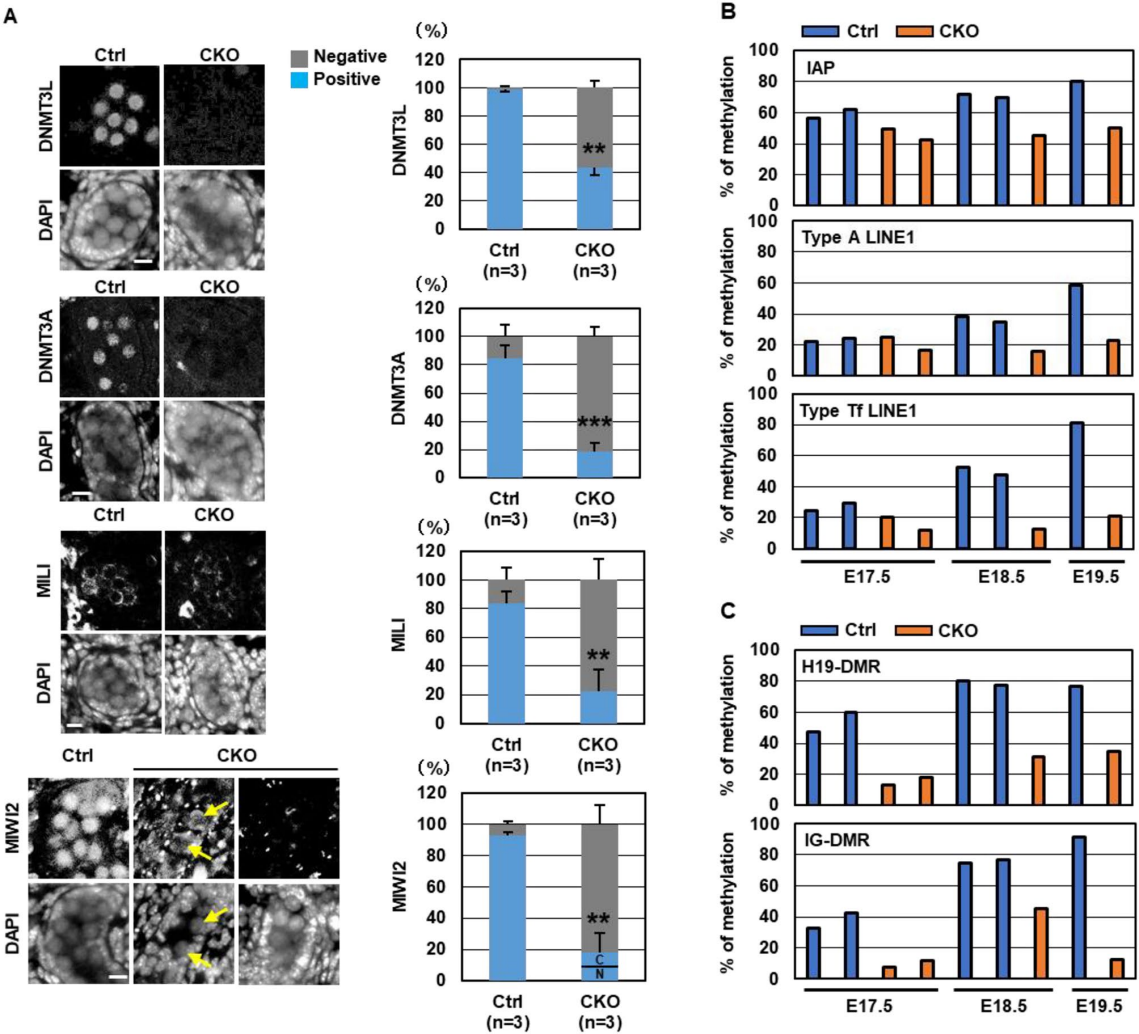
Impaired de novo DNA methylation in *Snf5* CKO male germ cells. (**A**) Immunohistochemistry of the E17.5 testes using antibodies against DNMT3L, DNMT3A, MILI, and MIWI2. Three mice were analyzed for each genotype, and the mean ± standard deviation is shown. The nuclei were counterstained with DAPI. The percentages of germ cells expressing these proteins were significantly lower in the *Snf5* CKO males (**P < 0.01, ***P < 0.001, Student’s *t*-test). Note that MIWI2 was absent from the nuclei and localized in the cytoplasm in some germ cells of the *Snf5* CKO males (yellow arrows). *C* cytoplasmic localization; *N* nuclear localization. Bar: 10 µm. (**B**,**C**) The DNA methylation status of retrotransposons and imprinted genes in male germ cells. The DNA methylation levels of IAP, LINE-1 (type A, type Tf) (**B**), and H19-DMR and IG-DMR (**C**) were examined by BS-Seq analyses using the purified germ cells at E17.5–E19.5.

RNA-seq and GO analyses revealed the lower expression of genes related to de novo DNA methylation in *Snf5* CKO males (Fig. 3C,G). In addition to mitotic arrest, de novo DNA methylation is a hallmark of fetal male germ cell differentiation. Therefore, we sought to clarify the protein expression of de novo DNA methyltransferases, DNMT3A and DNMT3L, and the central players involved in piRNA-mediated gene silencing, PIWIL2 (MILI), PIWIL4 (MIWI2), and MAEL in the testes of *Snf5* CKO males at E17.5. Consistent with the gene expression analyses, immunohistochemistry revealed that germ cells expressing DNMT3L, DNMT3A, MILI, MIWI2, and MAEL were significantly reduced in *Snf5* CKO males (Fig. 5A, Fig. S4A,B). Strong nuclear staining of MIWI2 was observed in the prospermatogonia of the control mice. However, in the *Snf5* CKO males, only approximately 20% of the germ cells expressed MIWI2, which was absent from the nuclei and localized in the cytoplasm in 40% of these MIWI2-positive cells (Fig. 5A). It is likely that the components involved in piRNA biogenesis may be expressed at lower levels in MIWI2-expressing cells (Fig. 3G) because piRNA biogenesis is required for the nuclear localization of MIWI2^35–37^. Moreover, the number of MVH-stained granules were reduced in the *Snf5* CKO mice, as observed in the *Mili* KO mice (Fig. S4C)^35^.

To investigate the impacts of the lower expression of these genes, we focused on the DNA methylation status in retrotransposons such as IAP, type A LINE1, and type Tf LINE1, as well as differentially methylated regions (DMRs) of the paternally imprinted genes, namely, H19-DMR and IG-DMR. Bisulfite sequencing (BS-Seq) analyses revealed that the percentage of methylation of these retrotransposons and imprinted genes gradually increased in the control germ cells from E17.5 to E19.5 (Fig. 5B,C, and Fig. S5). However, all of these regions were hypomethylated in *Snf5* CKO male germ cells, indicating that *Snf5* is involved in de novo DNA methylation in prospermatogonia via upregulation of de novo DNA methylation-related genes.

### Oogenesis and spermatogenesis in adult *Snf5* CKO mice

**Figure 6:**
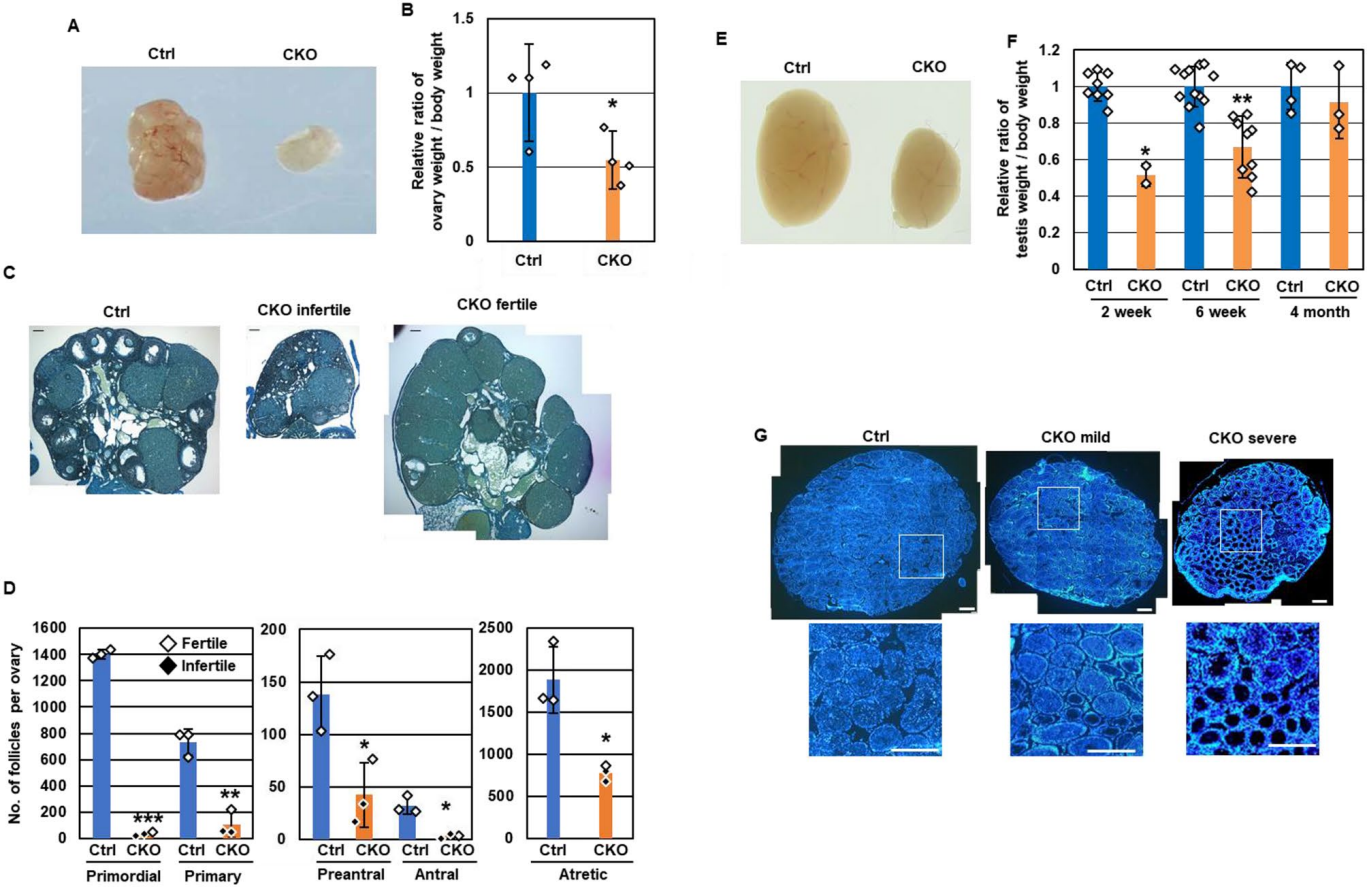
The ovaries and testes of adult *Snf5* CKO mice. (**A**) Representative photos of the ovaries of 6-week-old *Snf5* CKO mice. (**B**) Ratios of ovary weight per body weight in 6-week-old mice. The ratios were significantly lower in female *Snf5* CKO mice (*P < 0.05, Student’s *t*-test). The values from individual mice and the mean ± standard deviation are shown. (**C**) Histology of the ovaries from 4-month-old, infertile and fertile *Snf5* CKO females. The sections were stained with hematoxylin and picric methyl blue. Bars: 100 µm. (**D**) The number of follicles per ovary. The number of all the stages of follicles was significantly lower in the *Snf5* CKO females (*P < 0.05, **P < 0.005, ***P < 0.00005, Student’s *t*-test). The values from individual mice and the mean ± standard deviation are shown. Open and closed diamonds represent fertile and infertile mice, respectively. (**E**) Representative photos of the testes of 6-week-old *Snf5* CKO mice. (**F**) Ratios of testis weight per body weight in 2-week-old, 6-week-old and 4-month-old mice. The ratios were significantly lower in male *Snf5* CKO mice (*P < 5.0 × 10^−5^, **P < 0.0005, Student’s *t*-test). The values from individual mice and the mean ± standard deviation are shown. (**G**) Histology of the testes of 6-week-old, *Snf5* CKO males showing severe and mild phenotypes. Nuclei are counterstained with DAPI. Lower photos represent the higher magnification views of the enclosed areas. Bars: 300 µm.

Finally, we examined the fertility of adult *Snf5* CKO female (*Snf5*^*fx/Δ*^ + *TNAP-Cre*) by crossing to wild-type males. Four out of nine *Snf5* CKO females were infertile whereas the remaining five females gave birth to pups; the number of pups in the fertile *Snf5* CKO females was significantly lower than that in control mice (7.8 ± 1.1 (Control) vs 4.9 ± 2.0 (CKO), respectively, *P* < 0.001 by Student’s *t*-test; Table S1A). Genotyping of the pups revealed that about half of the pups (13 out of 28 pups) inherited *Snf5*^*fx*^ allele from the *Snf5* CKO females, indicating that the mature oocytes were derived from the germ cells in which Cre-mediated recombination had not occurred during PGC differentiation. Macroscopic inspection revealed atrophic ovaries in the *Snf5* CKO females (Fig. 6A,B). Histological analyses showed that the number of all the stages of follicles, especially primordial follicles, was dramatically lower in both sterile and fertile *Snf5* CKO females (Fig. 6C,D).

In contrast, all the *Snf5* CKO males were fertile and sired normal number of offspring (Table S1B). About half of the pups (7 out of 16 pups) inherited *Snf5*^*fx*^ allele whereas the remaining half (9 out of 16pups) inherited *Snf5*^*Δ*^ allele from the *Snf5* CKO males, suggesting that sperm were produced from the germ cells where the recombination had not taken place. Indeed, a majority of germ cells in the tubules containing the elongated spermatid were positive for SNF5 in the *Snf5* CKO mice (Fig. S6A). In the *Snf5* CKO males, the size of the testes was significantly smaller at 2-week and 6-week-old after birth, but became comparable to that of control mice at 4-month-old (Fig. 6E,F). Although we observed a number of germ cell-less seminiferous tubules in severe cases of 6-week-old mutants, the elongating spermatids were consistently detected in all the mutants (Fig. 6G). Because spermatogenesis are arrested at meiotic prophase in the *Brg1* CKO males^28,29^, the *Snf5*-deficient male germ cells presumably also could not differentiate beyond pachytene stage. Consistently, the number of TUNEL-positive cells significantly increased in the 6-week-old *Snf5* CKO mice (Fig. S6B,C). Taken together, these results suggest that the male germ cells retaining *Snf5*^*fx*^ allele undergo spermatogenesis to produce the sperm in the *Snf5* CKO males after birth.

## Discussion

In this study, we demonstrated that loss of *Snf5* in PGCs caused aberrant transition from sexually undifferentiated PGCs to female or male germ cells. In *Snf5* CKO females, both initiation and progression of meiosis were impaired. In *Snf5* CKO males, entry to mitotic arrest was delayed, and de novo DNA methylation was not acquired in retrotransposons or paternally imprinted genes. The developmental abnormalities were caused by failure not only to downregulate the PGC genes but also to upregulate female- or male-specific germline genes, indicating that the SWI/SNF complex is a prerequisite for sex-dependent transcriptional reprogramming in both sexes.

The phenotypic variations were observed in the individual *Snf5* CKO mice. For example, about half of the *Snf5* CKO females were sterile whereas the other half were fertile (Fig. 6C). Histological analyses of testes of the 6-week-old *Snf5* CKO males also showed variable spermatogenesis progression among seminiferous tubules and among individuals (Fig. 6G). Additionally, some germ cells underwent apoptosis during fetal stages but others did after birth in the *Snf5* CKO males (Fig. S2, S6). This is probably due to the variations in the efficiency and the timing of Cre-mediated recombination in the individual *TNAP-Cre* mice^31^. Actually, we also observed the variable number of SNF5-positive germ cells in the individual *Snf5* CKO mice (Fig. S1D,E).

Several studies have shown the critical roles of SWI/SNF complex in germline development. Maternal deletion of *Brg1* causes developmental arrest at 2-cell stage, which is accompanied by reduction of zygotic genome activation^27^. The deletions of *Brg1* in adult testes leads to arrest of spermatogenesis at prophase of meiosis I^28,29^. The spermatocytes of the *Brg1* CKO mice show asynapsis, accumulation of unrepaired DSBs and subsequent apoptosis. Similar meiotic defects were observed in the fetal oocytes of the *Snf5* CKO females (Fig. 4, S2A,B). Analyses of transcriptome of the *Brg1* CKO spermatogenic cells show the defective induction of meiosis-related genes^38^, indicating that SWI/SNF complex is required for activation of meiosis-related transcriptional programs in both sexes. Intriguingly, it has been reported that BRG1 associates with SCML, a testis-specific PRC1 component, and represses pre-meiotic and somatic genes, further promoting meiotic progression^38^. As the genes involved in PGC differentiation were not fully downregulated in *Snf5* CKO female and male germ cells at E15.5 (Fig. S3H,I). it would be interesting to examine whether SWI/SNF complex is involved in the repression of the PGC differentiation-genes in the future study.

Two transcriptional regulators, ZGLP1 and the STRA8–MEIOSIN complex, bind directly to the promoters and enhancers of a large number of meiosis-related genes to activate transcription^6,8,9,11^. *Meiosin*, but not *Zglp1* or *Stra8*, was downregulated in *Snf5* CKO female germ cells. It is likely that downregulation of the STRA8–MEIOSIN complex contributes to defective activation of meiosis-related genes. Consistently, 73% of the downregulated genes in *Stra8*-deficient oocytes were also downregulated in *Snf5* CKO cells (Fig. S7A,B). On the other hand, despite normal upregulation of *Zglp1*, the ZGLP1 target genes were also downregulated in *Snf5* CKO germ cells (Fig. S7B). As remodelers are targeted to promoters and enhancers cooperatively by sequence-specific transcription factors^19,20^, the SWI/SNF complex may be recruited by ZGLP1 and the STRA8–MEIOSIN complex to activate meiosis-related genes.

The SWI/SNF complex evicts PRCs from chromatin harboring H3K27me3 marks to activate gene expression^21–23^. It has been reported that PRC1 represses meiosis-related genes in female PGCs before entering meiosis^12^. A previous study showed that H3K27me3 and the bivalent histone modifications were enriched in the promoters of 44% and 14% of meiosis-related genes, respectively, in PGC-like cells before sex differentiation (Fig. S7C)^11^. Although ZGLP1 target genes were enriched among genes with H3K27me3 marks^11^, the downregulated genes in *Snf5* CKO germ cells included genes with H3K4me3 marks as well as genes with H3K27me3 marks (Fig. S7C)^39^. However, since 81% of the ZGPL1 target genes were downregulated in *Snf5* CKO cells (Fig. S7B), it is possible that the SWI/SNF complex may replace PRCs to activate genes with H3K27me3 marks in the germ cells initiating female differentiation.

In contrast to females, the transcriptional mechanisms that initiate and drive male germ cell differentiation remain unclear. We consider that similar to female germ cells, the SWI/SNF complex may activate male-specific germline genes cooperatively with specific transcription factors. Further studies will be required to reveal the mechanisms by which the SWI/SNF complex reprograms the transcriptome for the initiation of sex-dependent germ cell differentiation. Finally, our studies show that the SWI/SNF chromatin remodeling complex involves sex-specific differentiation in both female and male germ cells.

## Methods

### Mice

Mice were housed in a specific pathogen-free, temperature-controlled facility with a 12-h light/dark cycle in individual ventilated cages. Animals were provided food and water ad libitum. All animal care and experimental procedures were performed in accordance with the Guidelines of Animal Experiments of Kitasato University and were approved by the Institutional Animal Care and Use Committee of Kitasato University (Approval number: SAS2004). The floxed *Snf5* mice, *TNAP-Cre* mice, and *Oct4-EGFP* mice were described previously^30–32^. We used the mice with mixed genetic background (C57BL/6, 129/Sv, and ICR). The mouse genotypes were determined as summarized in Table S2. The study was carried out in compliance with the ARRIVE guidelines.

### Immunohistochemistry

To prepare frozen sections, dissected gonads with mesonephros were fixed with 4% paraformaldehyde in phosphate buffered saline (PBS) for 3 h to overnight. The fixed gonad material was washed two times in PBS at 4 °C for at least 1 h and then saturated in a sucrose series: 10% (1 h) followed by 20% (overnight) at 4 °C. The gonads were then embedded in OCT compound (Sakura, Tokyo, Japan), frozen in liquid nitrogen and cut at 7 μm thickness. Antigen retrieval was performed for all antibodies; the slides were autoclaved for 20 min at 120 °C or microwaved for 15 min in citrate buffer (10 mM citric acid, pH 6.0). After 30 min, the slides were washed three times in PBS for 3 min. The sections were permeabilized with 0.1% Triton X-100 in PBS for 6 min and washed three times in PBS for 1 min. The sections were blocked for 1 h at 4 °C in PBS containing 10% Blocking One (Nacalai, Kyoto, Japan) and then incubated with primary antibodies in blocking solution overnight at 4 °C. The slides were washed three times for 1 min in PBS. The sections were then incubated with secondary antibodies for 2 h at room temperature. The slides were washed four times for 1 min in PBS and one time in distilled water. The sections were mounted using 10% DABCO and 50% glycerol in PBS. Immunofluorescence was observed using a BX51 fluorescence microscope (Olympus, Tokyo, Japan), and photographs were taken using a DP73 digital CCD camera (Olympus). Adobe Photoshop Elements 4.0 (Adobe, San Jose, CA, USA) was used for image editing. The number of germ cells was counted in the photos. The antibodies used are listed in Table S3. The nuclei were counterstained with 1 µg/ml 4',6-diamidino-2-phenylindole (DAPI) or 5 μg/ml Hoechst 33,342. For SNF5 staining, TBST (0.05 mol/L Tris–HCl, 0.15 mol/L NaCl, 0.05% Tween20) was used for wash buffer. For frozen sections (fetal ovaries and testes), antigen retrieval was done by microwave 15 min in citrate buffer (10 mM citric acid, pH 6.0). For paraffin sections (adult testes), antigen retrieval was done by autoclaved for 20 min at 120 °C in EDTA buffer (1 mM EDTA, 0.05% Tween 20, pH 8.0). The sections were blocked for 1 h at 4 °C in 0.05 mol/L Tris–HCl buffer with 1% BSA and then incubated with primary antibodies in blocking solution 1 h at room temperature^40–42^. The signal of SNF5 was detected with anti-mouse Envision + kit (DAKO, Carpinteria, CA) and developed by using a diaminobenzidine chromogen (Nacalai).

### Quantification of fetal germ cells

The number of PGCs at E12.5 and E13.5 were determined by counting the SSEA-1-positive cells by flow cytometry. The number of germ cells at E15.5–E19.5 was determined as follows. The sections were stained with anti-MVH antibody as described above. First, the sections with maximum number of germ cells were selected and then the adjacent sections were collected ever other section to avoid counting the same cells. The number of germ cells with clear nuclei were counted in more than 3 sections per mouse and the average numbers are shown.

### Follicle count

Ovaries were fixed (0.34 N glacial acetic acid, 10% formalin, 28% ethanol), paraffin embedded, serially sectioned (8 μm), and stained with hematoxylin and picric methyl blue. Classification and counting of follicles were carried out as described previously^43,44^. Briefly, the number of primordial, primary and atretic follicles in every fifth section was determined. Total number of follicles per ovary was estimated by multiplying the cumulative counts by a correction factor of five. The number of secondary and antral follicles was estimated by exact counts determined from whole sections of ovary.

### Chromatin spread analysis

The chromatin spreads were prepared using the E19.5 ovaries as described previously, except we used Drywell (Fujifilm, Tokyo, Japan) instead of Photoflo^45^. Immunohistochemistry and image editing were performed as described above. The antibodies used in the chromatin spread are listed in Table S4. The nuclei were counterstained with 1 μg/ml DAPI.

### Fluorescence-activated cell sorting analysis (FACS)

Dissected gonads without mesonephros were treated in 0.01% Trypsin/EDTA in PBS for 10 min at 37 °C. The single cell suspension was fixed with 4% paraformaldehyde in PBS for 15 min at room temperature. washed in PBS for one time and incubated on ice in mixed solution containing primary (SSEA1) and secondary antibodies in 10% blocking one in PBS for 30 min. The cells were washed two times in PBS and replaced 1% FCS in PBS. FACS were done by using Gallios (Beckman Coulter, Brea, CA, USA). The results were analyzed using Kaluza v1.5 or v2.0 (Beckman Coulter, Brea, CA, USA).

### Isolation of germ cells expressing the Oct4-EGFP transgene by FACS

Germ cells expressing *Oct4-EGFP* (EGFP under the control of the *Oct4* promoter) were isolated from ovaries and testes using Moflo XDP (Beckman Coulter, Brea, CA, USA). The number and purity of the germ cells are summarized in Table S4.

### RNA-Seq analysis

RNA was isolated using the RNeasy Micro Kit (Qiagen, Venlo, Netherlands). RNA-Seq samples were prepared in biological duplicates (Table S5). The cDNA was synthesized from 10 ng total RNA and was amplified using the SMART-Seq v4 Ultra Low Input RNA Kit for Sequencing (Clontech, Palo Alto, CA, USA). RNA-seq libraries were prepared from the amplified cDNA (150 pg for each sample) using the Nextera XT Kit (Illumina, San Diego, CA, USA). Paired-end 75 or 100 bp sequencing was conducted on the HiSeq 2500 using the HiSeq SBS Kit v4 – HS, or on the NextSeq 500 platform using the NextSeq 500/550 High Output Kit v2.5 (Illumina, San Diego, CA, USA). The obtained sequence data were converted to FASTQ files using bcl2fastq conversion software (Illumina, San Diego, CA, USA).

Published sequence datasets were downloaded using the SRA-Toolkit (NIH). RNA-seq data were analyzed using Tophat v2.1.1 (https://ccb.jhu.edu/software/tophat/index.shtml), Cufflinks v2.2.1 (http://cole-trapnell-lab.github.io/cufflinks/). We downloaded UCSC mm10 as a reference genome. Heatmaps and PCA were conducted using R (version 3.4.2)^46^ and the R packages pheatmap and prcomp. GO analysis was performed using DAVID (https://david.ncifcrf.gov/summary.jsp). We constructed an automated analysis system from the downloaded SRA file to calculation of FPKM using Cufflinks. Details are available upon request. Our RNA-seq datasets are filed under accession number GSE154209. The RNA-seq analyses are filed under accession numbers GSE76973^5^, GSE94136^4^, and GSE124262^11^.

### BS-Seq analysis

The sorted cells were lysed using 2 mg/ml Proteinase K solution together with 500 ng salmon sperm DNA. The denatured DNA was incubated in bisulfite reaction buffer (4.25 M Na_2_S_2_O_5_, 0.117 M hydroquinone, 0.28 M NaOH, and 21% tetrahydrofurfuryl alcohol) at 50 °C for 4 h and with desulphonation buffer (0.2 M NaOH, 80% ethanol) at 37 °C for 15 min. After the addition of 500 ng salmon sperm DNA, the DNA was isolated using a Zymo-Spin IC column (Zymo Research, Irvine, CA, USA). The first and second rounds of polymerase chain reaction (PCR) were conducted using KOD Multi & Epi DNA polymerase (Toyobo, Osaka, Japan). The PCR conditions were as follows: in the first round, 2 min at 94 °C followed by 35 cycles of 10 s at 98 °C, 30 s at 50 °C, and 30 s at 68 °C, and a final 1 min at 68 °C; in the second round, 3 min at 95 °C followed by 8 cycles of 30 s at 95 °C, 30 s at 55 °C, and 30 s at 72 °C, and a final 5 min at 72 °C. The primer sequences used in the first PCR are listed in Table S6. The primers with barcodes provided by Illumina were used in the second PCR. The PCR products were purified using NucleoMag beads (Takara, Shiga, Japan) and sequenced using the MiSeq system (Illumina). The sequences were analyzed using QUMA-San, a modified version of QUMA (http://quma.cdb.riken.jp/top/quma_main_j.html). Details are available upon request.

## Supplementary Information

Supplementary Information.

## Data Availability

The accession number of RNA-Seq analysis is GSE154209. The datasets used and/or analyzed during the current study are available from supplementary information and the corresponding author on reasonable request.

## References

[1] Ginsburg M, Snow MHL, Mclaren A (1990). Primordial germ cells in the mouse embryo during gastrulation. Development.

[2] Lawson KA (1999). Bmp4 is required for the generation of primordial germ cells in the mouse embryo. Genes Dev..

[3] Spiller C, Koopman P, Bowles J (2017). Sex determination in the mammalian germline. Annu. Rev. Genet..

[4] Miyauchi H (2017). Bone morphogenetic protein and retinoic acid synergistically specify female germ-cell fate in mice. EMBO J..

[5] Hill PWS (2018). Epigenetic reprogramming enables the transition from primordial germ cell to gonocyte. Nature.

[6] Soh YQS (2015). A gene regulatory program for meiotic prophase in the fetal ovary. PLoS Genet..

[7] Lesch BJ, Page DC (2012). Genetics of germ cell development. Nat. Rev. Genet..

[8] Ishiguro K (2020). MEIOSIN directs the switch from mitosis to meiosis in mammalian germ cells. Dev. Cell.

[9] Kojima ML, De Rooij DG, Page DC (2019). Amplification of a broad transcriptional program by a common factor triggers the meiotic cell cycle in mice. Elife.

[10] Vernet N (2020). Meiosis occurs normally in the fetal ovary of mice lacking all retinoic acid receptors. Sci. Adv..

[11] Nagaoka SI (2020). ZGLP1 is a determinant for the oogenic fate in mice. Science.

[12] Yokobayashi S (2013). PRC1 coordinates timing of sexual differentiation of female primordial germ cells. Nature.

[13] Bowles J (2006). Retinoid signaling determines germ cell fate in mice. Science.

[14] Suzuki A, Saga Y (2008). Nanos2 suppresses meiosis and promotes male germ cell differentiation. Genes Dev..

[15] Suzuki A, Igarashi K, Aisaki KI, Kanno J, Saga Y (2010). NANOS2 interacts with the CCR4-NOT deadenylation complex and leads to suppression of specific RNAs. Proc. Natl. Acad. Sci. USA.

[16] Saba R, Kato Y, Saga Y (2014). NANOS2 promotes male germ cell development independent of meiosis suppression. Dev. Biol..

[17] Hata K, Okano M, Lei H, Li E (2002). Dnmt3L cooperates with the Dnmt3 family of de novo DNA methyltransferases to establish maternal imprints in mice. Development.

[18] Kato Y (2007). Role of the Dnmt3 family in de novo methylation of imprinted and repetitive sequences during male germ cell development in the mouse. Hum. Mol. Genet..

[19] Kassabov SR, Zhang B, Persinger J, Bartholomew B (2003). SWI/SNF unwraps, slides, and rewraps the nucleosome. Mol. Cell.

[20] Bracken AP, Brien GL, Verrijzer CP (2019). Dangerous liaisons: Interplay between SWI/SNF, NURD, and polycomb in chromatin regulation and cancer. Genes Dev..

[21] Schuettengruber B, Bourbon HM, Di Croce L, Cavalli G (2017). Genome regulation by polycomb and trithorax: 70 years and counting. Cell.

[22] Kadoch C (2017). Dynamics of BAF-polycomb complex opposition on heterochromatin in normal and oncogenic states. Nat. Genet..

[23] Nakayama RT (2017). SMARCB1 is required for widespread BAF complex-mediated activation of enhancers and bivalent promoters. Nat. Genet..

[24] Mittal P, Roberts CWM (2020). The SWI/SNF complex in cancer—biology, biomarkers and therapy. Nat. Rev. Clin. Oncol..

[25] Kim KH (2015). SWI/SNF-mutant cancers depend on catalytic and non-catalytic activity of EZH2. Nat. Med..

[26] Klochendler-Yeivin A (2000). The murine SNF5/INI1 chromatin remodeling factor is essential for embryonic development and tumor suppression. EMBO Rep..

[27] Bultman SJ (2006). Maternal BRG1 regulates zygotic genome activation in the mouse. Genes Dev..

[28] Kim Y, Fedoriw AM, Magnuson T (2012). An essential role for a mammalian SWI/SNF chromatin-remodeling complex during male meiosis. Development.

[29] Wang J, Gu H, Lin H, Chi T (2012). Essential roles of the chromatin remodeling factor Brg1 in spermatogenesis in mice. Biol. Reprod..

[30] Roberts CWM, Leroux MM, Fleming MD, Orkin SH (2002). Highly penetrant, rapid tumorigenesis through conditional inversion of the tumor suppressor gene Snf5. Cancer Cell.

[31] Lomelí H, Ramos-Mejía V, Gertsenstein M, Lobe CG, Nagy A (2000). Targeted insertion of Cre recombinase into the TNAP gene: excision in primordial germ cells. Genesis.

[32] Yoshimizu T (1999). Germline-specific expression of the Oct-4/green fluorescent protein (GFP) transgene in mice. Dev. Growth Differ..

[33] Lin Y, Gill ME, Koubova J, Page DC (2008). Germ cell-intrinsic and -extrinsic factors govern meiotic initiation in mouse embryos. Science.

[34] Gill ME, Hu YC, Lin Y, Page DC (2011). Licensing of gametogenesis, dependent on RNA binding protein DAZL, as a gateway to sexual differentiation of fetal germ cells. Proc. Natl. Acad. Sci. USA.

[35] Kuramochi-Miyagawa S (2010). MVH in piRNA processing and gene silencing of retrotransposons. Genes Dev..

[36] Yoshimura T (2018). Mouse GTSF 1 is an essential factor for secondary pi RNA biogenesis. EMBO Rep..

[37] Shiromoto Y (2019). GPAT2 is required for piRNA biogenesis, transposon silencing, and maintenance of spermatogonia in mice. Biol. Reprod..

[38] Menon, D. U., Shibata, Y., Mu, W. & Magnuson, T. Mammalian SWI/SNF collaborates with a polycomb-associated protein to regulate male germline transcription in the mouse. *Dev.* **146**, (2019). 10.1242/dev.174094 PMC6803380 31043422

[39] Ohta H (2017). In vitro expansion of mouse primordial germ cell-like cells recapitulates an epigenetic blank slate. EMBO J..

[40] DelBove J (2009). Inactivation of SNF5 cooperates with p53 loss to accelerate tumor formation in Snf5+/−; p53 +/− mice. Mol. Carcinog..

[41] Kuwahara Y (2013). Establishment and characterization of MRT cell lines from genetically engineered mouse models and the influence of genetic background on their development. Int. J. Cancer.

[42] Ng JMY (2015). Generation of a mouse model of atypical Teratoid/ Rhabdoid tumor of the central nervous system through combined deletion of Snf5 and p53. Cancer Res..

[43] Myers M, Britt KL, Wreford NGM, Ebling FJP, Kerr JB (2004). Methods for quantifying follicular numbers within the mouse ovary. Reproduction.

[44] Johnson J, Canning J, Kaneko T, Pru JK, Tilly JL (2004). Germline stem cells and follicular renewal in the postnatal mammalian ovary. Nature.

[45] Peters AHFM, Plug AW, Van Vugt MJ, De Boer P (1997). A drying-down technique for the spreading of mammalian melocytes from the male and female germline. Chromosome Res..

[46] R Core Team. R: A language and environment for statistical computing. R Foundation for Statistical Computing, Vienna, Austria. URL https://www.R-project.org/ (2017).

