# Supplementary material for: SWI/SNF chromatin remodeling complex is required for initiation of sex-dependent differentiation in mouse germline
Source: Sci Rep. 2021 Dec 15;11:24074. doi: 10.1038/s41598-021-03538-8 (PMC8674328; doi:10.1038/s41598-021-03538-8)
Supplement: Supplementary file 1 — Supplementary Information. [file 41598_2021_3538_MOESM1_ESM.pdf]

SUPPLEMENTARY DATA

SWI/SNF chromatin remodeling complex is required for initiation of sex-dependent differentiation in mouse germline

Authors and affiliations:

Toshiaki Ito[1], Atsuki Osada[1], Masami Ohta[1], Kana Yokota[1], Akira Nishiyama[2], Yuichi Niikura[3], Tomohiko Tamura[2], Yoichi Sekita[1], and Tohru Kimura[1]

[1] Laboratory of Stem Cell Biology, Graduate School of Science, and Department of Biosciences, School of Science, Kitasato University, 1-15-1, Kitasato, Minami-ku, Sagamihara, Kanagawa 252-0373, Japan.

[2] Department of Immunology, Yokohama City University Graduate School of Medicine, 3-9 Fukuura, Kanazawa-ku, Yokohama, Kanagawa 236-0004, Japan.

[3] Faculty of Pharmaceutical Sciences, Josai International University, 1 Gumyo, Togane, Chiba, 283-8555, Japan.

Author contribution

T.I., A.O., M.O., K.Y., Y.N., and Y.S. performed the experiments. A. N. and T.T. performed RNA-Seq analyses. All the authors analyzed the data. T.I., A.O., Y.S. and T.K. designed the research and wrote the paper. All the authors approved the manuscript.

Correspondence:

* Tohru Kimura, PhD

Laboratory of Stem Cell Biology, Department of Biosciences, Kitasato University School of Science,

1-15-1 Kitasato, Minami-ku, Sagamihara, Kanagawa, 252-0373, Japan

Tel: +81-42-778-8864;   Fax: +81-42-778-8864

E-mail: tkimura@ kitasato-u.ac.jp

Supplementary Figures; Figure S1–S4.
Supplementary Tables; Table S1–S6.
Supplementary Methods
Supplementary References

Supplementary Figures

Ito_Figure S1

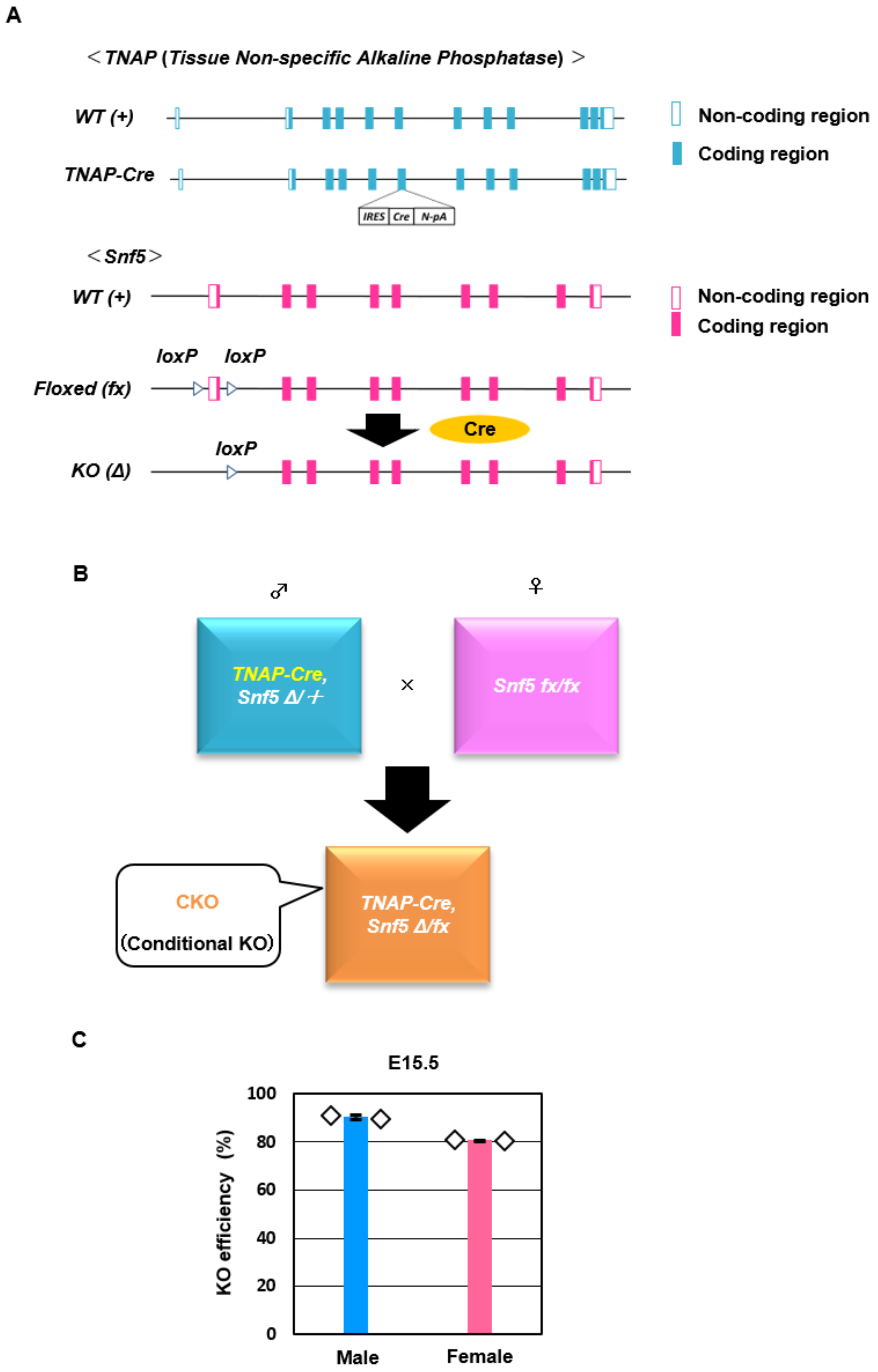

Ito_Figure S1

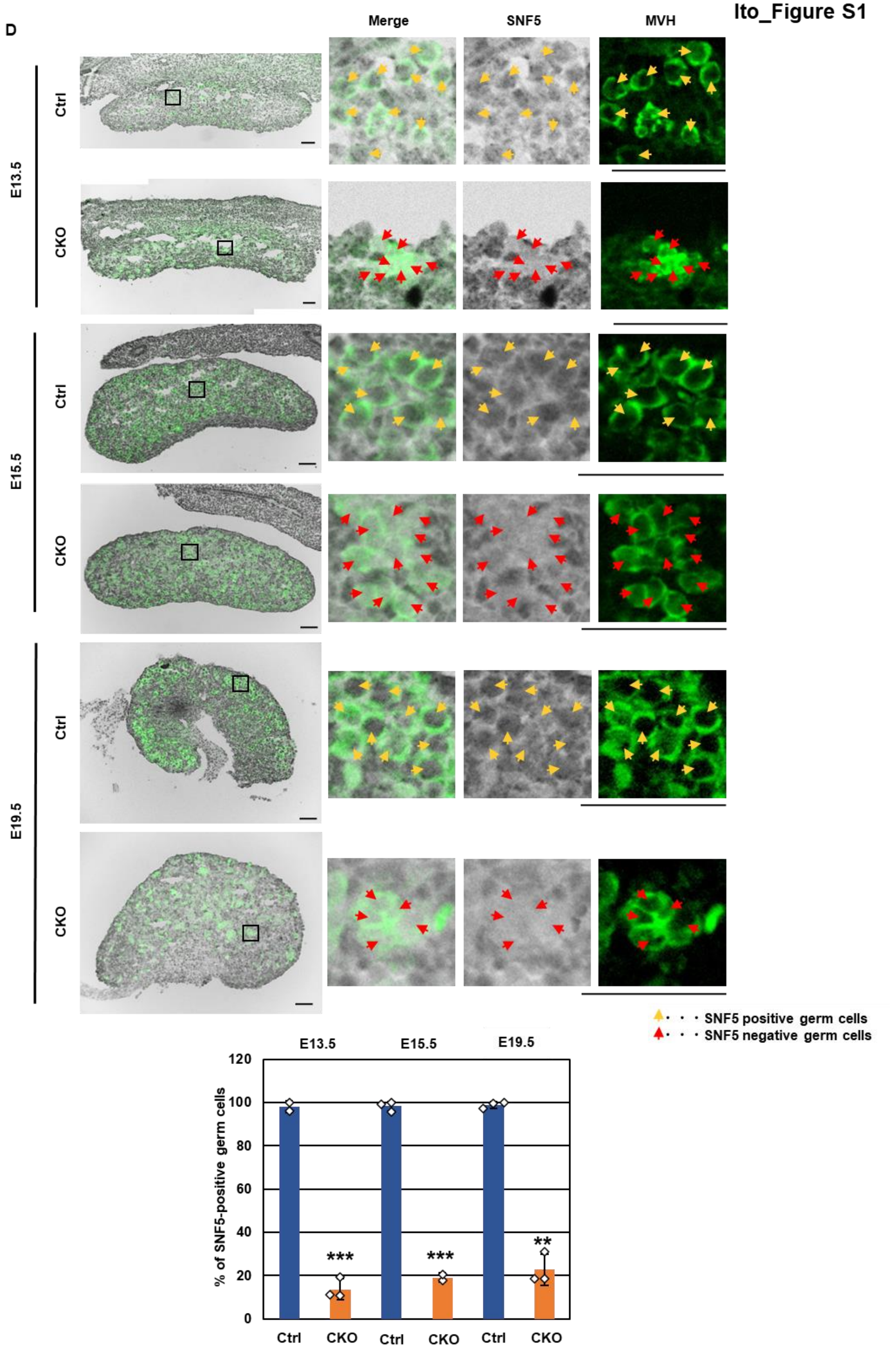

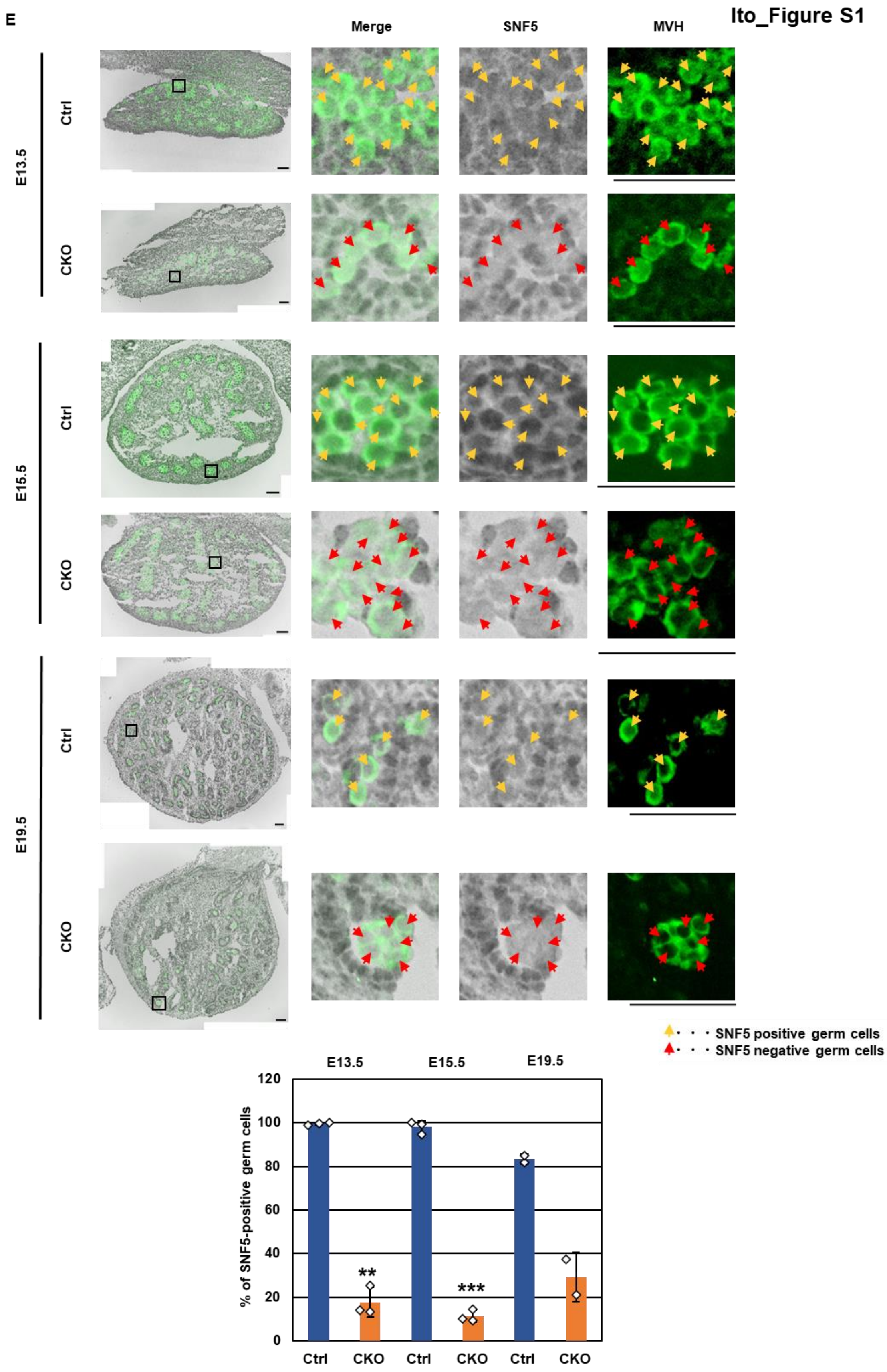
E
Ito_Figure S1
Merge
SNF5
MVH
E13.5
Ctrl
CKO
E15.5
Ctrl
CKO
E19.5
Ctrl
CKO
SNF5 positive germ cells
SNF5 negative germ cells
E13.5
E15.5
E19.5
% of SNF5-positive germ cells
120
100
80
60
40
20
0
**
***
Ctrl
CKO
Ctrl
CKO
Ctrl
CKO

Figure S1. Generation of PGC-specific *Snf5* CKO mice.

(A) Structures of the *TNAP-Cre* and floxed *Snf5* loci. At the *TNAP-Cre* locus, the *IRES-Cre* cassette was knocked into exon 6 of *TNAP* (*ALPL*). In the floxed *Snf5* locus, exon 1 encoding the start codon was flanked by two *loxP* sequences.

(B) Scheme to generate PGC-specific *Snf5* CKO mice. Heterozygous *Snf5* KO males ($Snf5^{+/\Delta}$) harboring the *TNAP-Cre* locus were crossed to homozygous floxed *Snf5* females ($Snf5^{fx/fx}$) to generate PGC-specific *Snf5* CKO mice ($Snf5^{fx/\Delta}$ + *TNAP-Cre*). Mice with other genotypes were used as the controls ($Snf5^{fx/+}$ mice, $Snf5^{fx/\Delta}$ mice, and $Snf5^{fx/+}$ mice harboring the *TNAP-Cre* allele). Mice carrying the *Oct4-EGFP* transgene were used to isolate germ cells.

(C) Cre-mediated recombination efficiencies at the *floxed Snf5* loci in E15.5 male and female germ cells. Using RNA-Seq data from E15.5 male and female germ cells, the reads that mapped to exon 1 of the *Snf5* loci were assessed. The recombination efficiencies were estimated as described in the Supplementary Methods.

(D, E) Immunohistochemistry using anti-SNF5 antibody. The sections of ovaries (D) and testes (E) at E13.5, E15.5 and E19.5 were immunostained with anti-SNF5 and anti-MVH antibodies. The inserts show higher-magnification views. The yellow and red arrowheads indicate SNF5-positive and the SNF5-negative germ cells, respectively. The percentages of SNF5-positive germ cells in individual mice are shown in bar graphs (the mean ± standard deviation; $^{**}P < 0.01$, $^{***}P < 0.001$, Student's *t*-test). Bars: 50 µm.

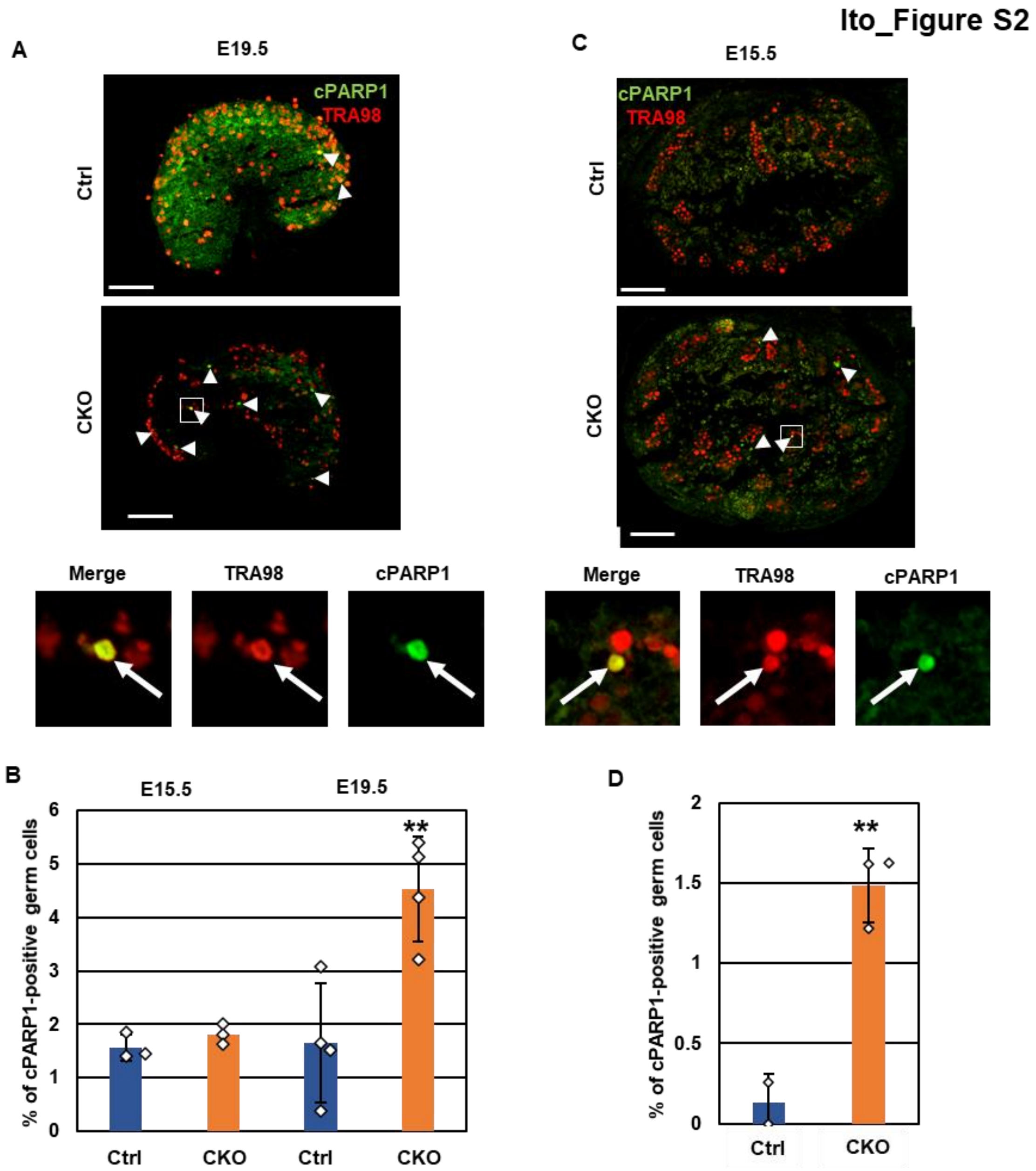

Figure S2 Apoptosis in the *Snf5* CKO mice.

(A, C) The cleaved PARP1 (cPARP1)-positive apoptotic germ cells in the *Snf5* CKO mice. The sections of the E19.5 ovaries (A) and the E15.5 testes (C) were immunostained with germ cell marker (TRA98) and apoptotic cell marker (cPARP1). The inserts show higher-magnification views. Arrowheads and arrows indicate apoptotic germ cells. Bars: 50 µm.

(B, D) The percentages of cPARP1-positive apoptotic germ cells in female (B) and male (D) mice. The values from individual mice and the mean ± standard deviation are shown (**$P < 0.01$, Student's *t*-test).

Ito_Figure S3

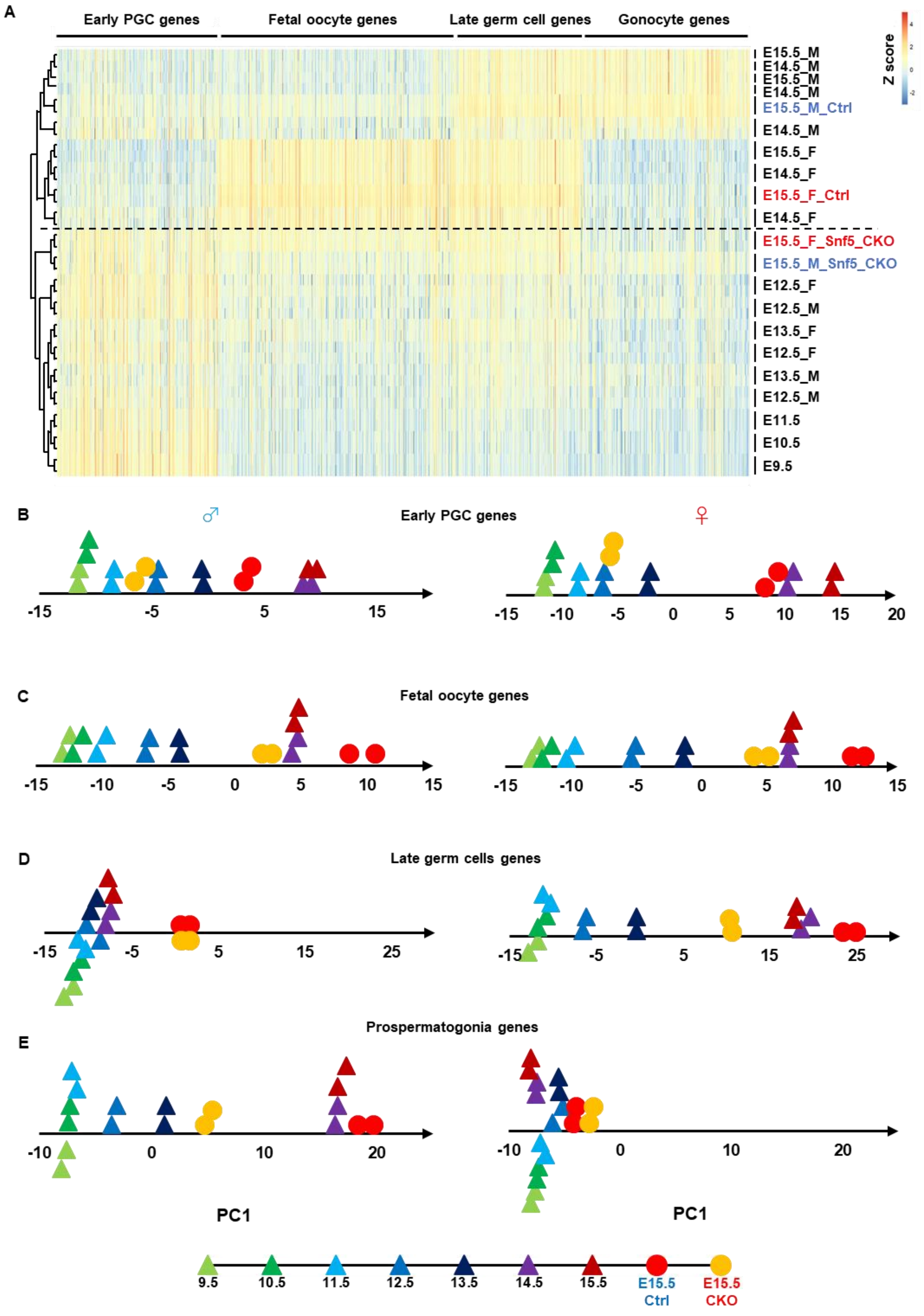

Ito_Figure S3

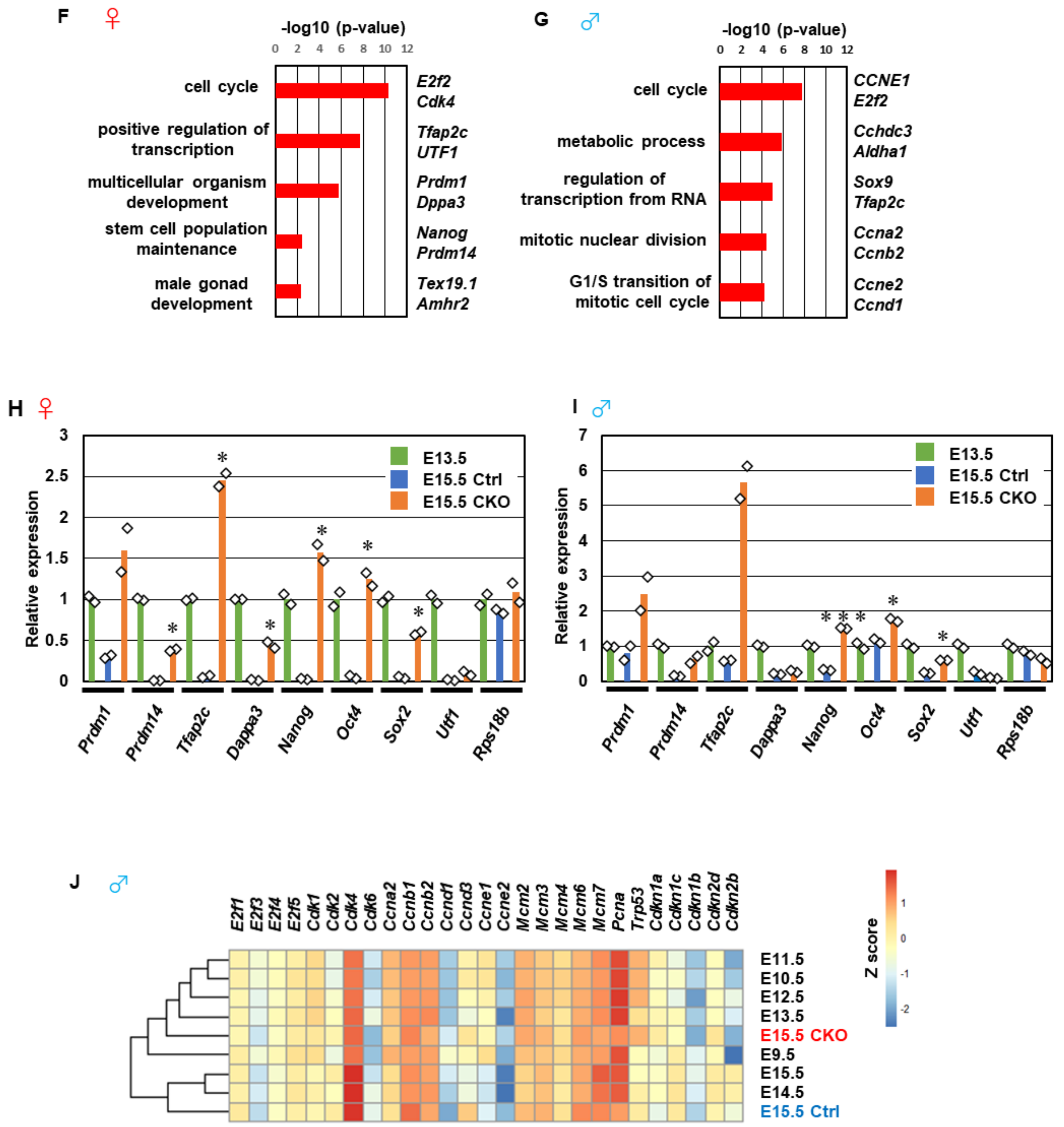

Figure S3. Gene expression analyses of female and male fetal germ cells from *Snf5* CKO mice.

(A) Hierarchical cluster analyses of the E15.5 control and *Snf5* CKO germ cells (female, red letters; male, blue letters). Early PGC genes (290 genes), fetal oocyte genes (430 genes), late germ genes (232 genes), and prospermatogonia genes (300 genes) have been described previously [1]. The expression of these genes in our RNA-Seq datasets from germ cells at E15.5 and in previous RNA-Seq datasets from germ cells at E9.5–E15.5 were analyzed.

(B–E) PCA of early PGC, fetal oocyte, late germ cell, and prospermatogonia genes.

(F, G) GO analyses of the genes whose expression levels were significantly higher in female (F) and male (G) *Snf5* CKO germ cells at E15.5.

(H, I) Expression of the key genes involved in early PGC differentiation in females (H) and males (I). The expression levels in control germ cells at E13.5 and E15.5 and in *Snf5* CKO germ cells at E15.5 are shown (the values of the E13.5 control cells are set as 1.0). The expression of *Rps18b* as the internal control is shown.

(J) PCA analyses of the growth-related genes.

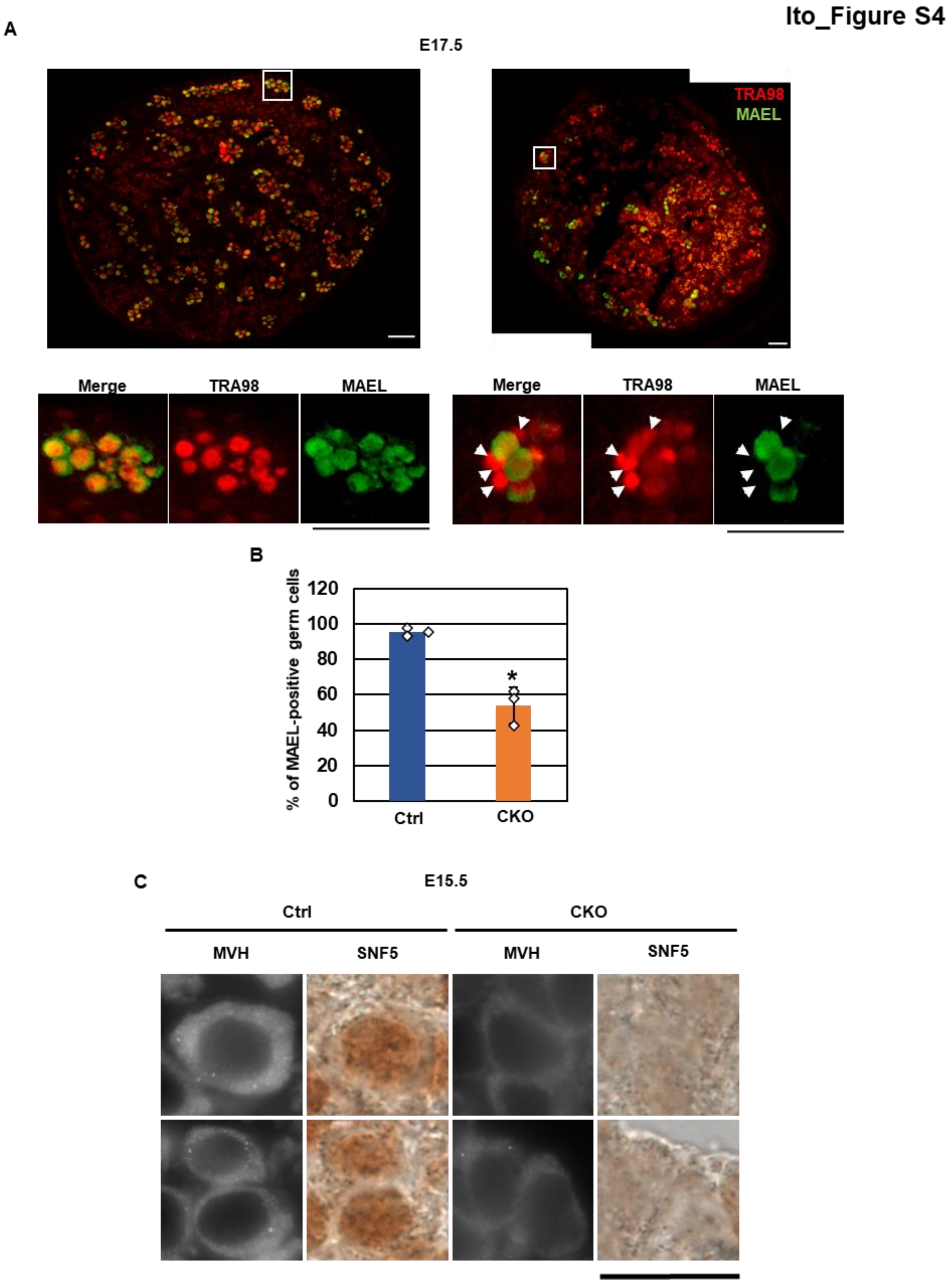

Figure S4 Immunohistochemistry using antibodies against MAEL and MVH

(A) The sections of the E17.5 testes were immunostained with anti-MAEL antibody and germ cell marker TRA98. The inserts show higher-magnification views. Arrowheads indicate MAEL-negative germ cells. Bars: 50 µm.

(B) The percentages of MAEL-positive germ cells. The values from individual mice and the mean ± standard deviation are shown (*$P < 0.05$, Student's *t*-test).

(C) The sections of the E17.5 testes were immunostained with antibodies against MAEL and SNF5. Note that the number of MVH-positive granules were reduced in SNF5-negative germ cells. Bars: 10 µm.

Ito_Figure S5

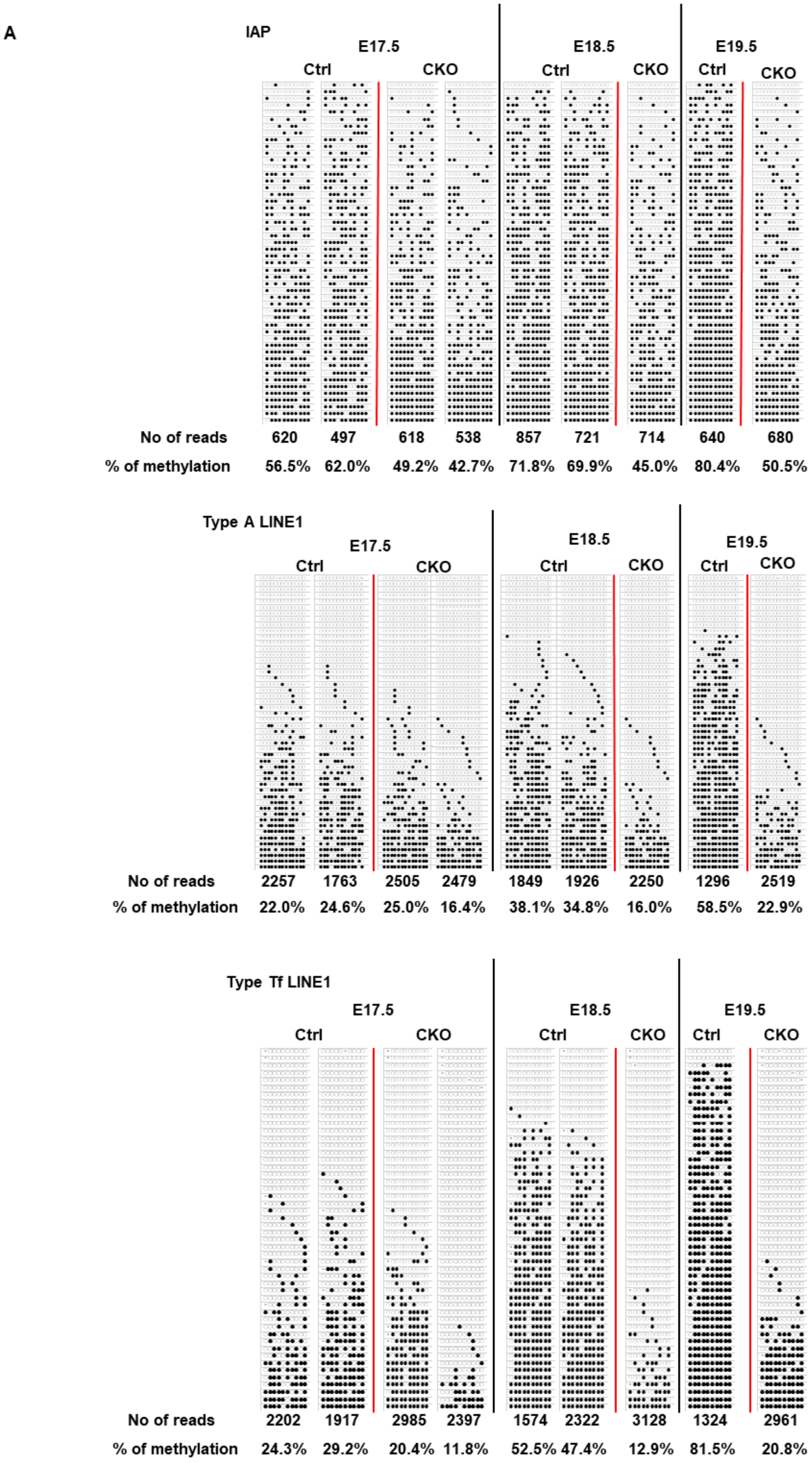

Ito_Figure S5

B

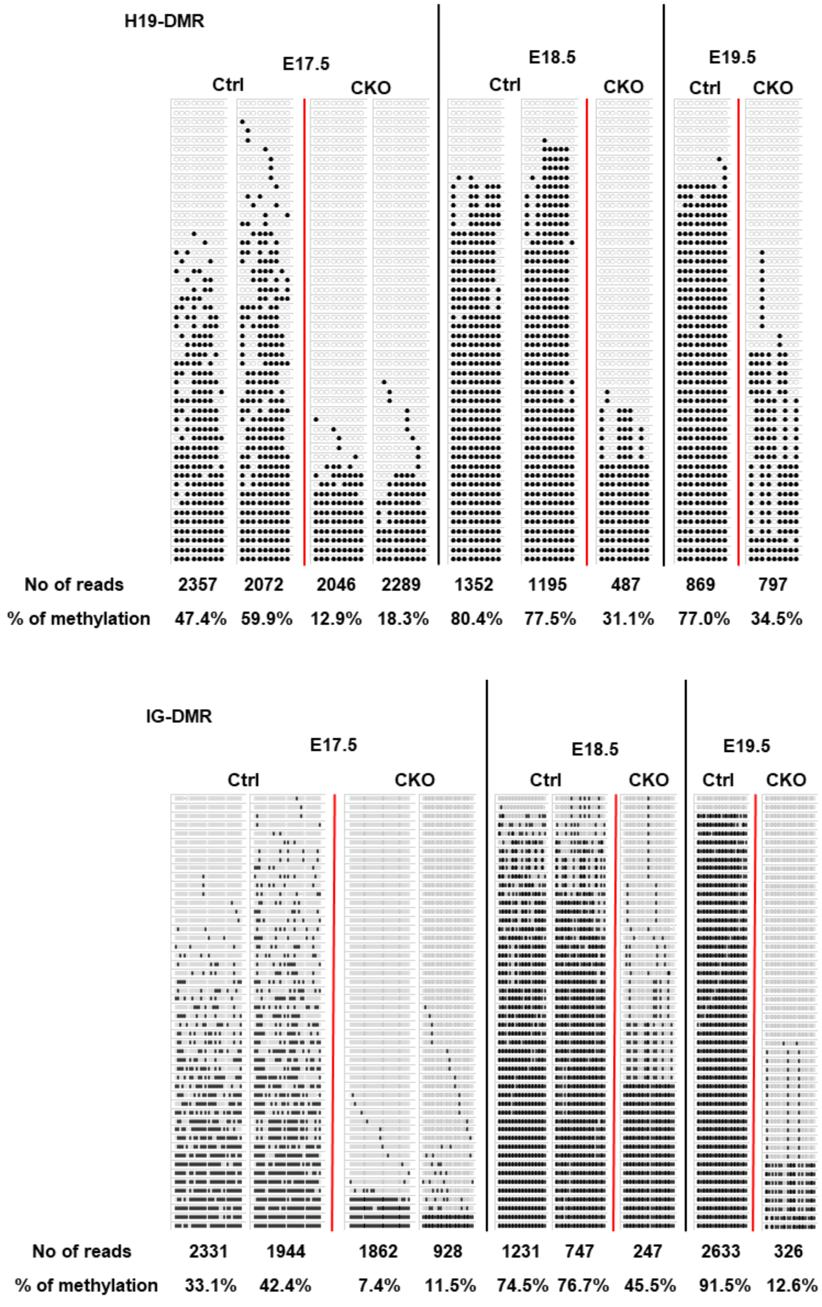

Figure S5. BS-Seq analyses.

The male germ cells isolated from control and *Snf5* CKO mice were subjected to BS-Seq analyses. The methylation levels of IAP and LINE1 (A) and H19-DMR and IG-DMR (B) were examined. Fifty representative reads are shown (closed circles, methylated CpG; open circles, unmethylated CpG). The total number of reads and methylation percentages in the total reads are shown.

Ito_Figure S6

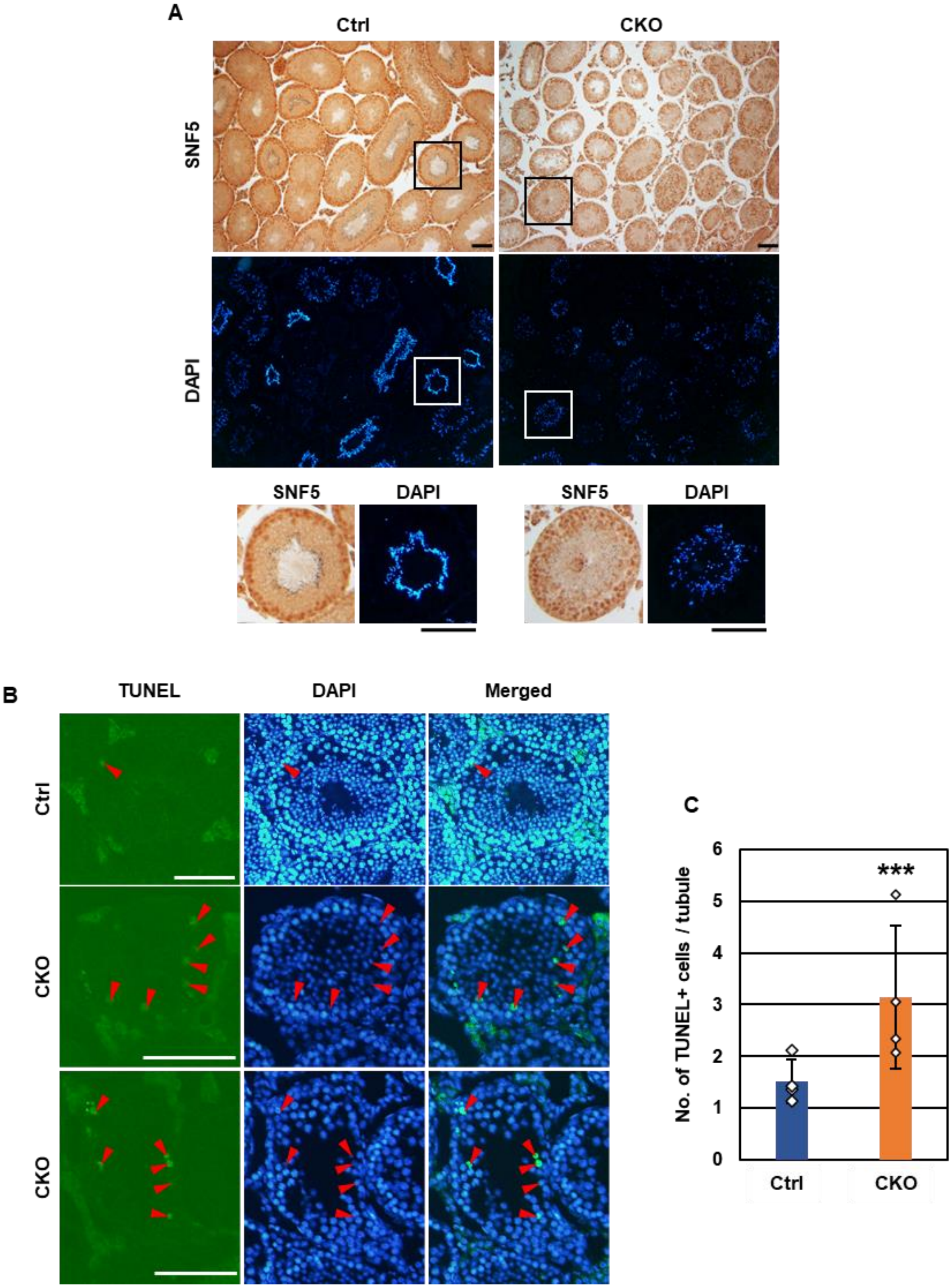

Figure S6 The testes of the 6-week-old *Snf5* CKO mice

(A) Immunohistochemistry using SNF5 antibody. The inserts show higher-magnification views. Note that a majority of germ cells in the tubule containing elongated spermatids were positive for SNF5. Nuclei were counterstained with DAPI. Bars: 100 μm.

(B) TUNEL assay. The red arrowheads indicate TUNEL-positive cells. Nuclei were counterstained with DAPI. Bars: 100 μm.

(C) The percentages of TUNEL-positive cells per tubule. The values from individual mice and the mean ± standard deviation are shown (***$P < 0.0001$, Student's *t*-test).

Ito_Figure S7

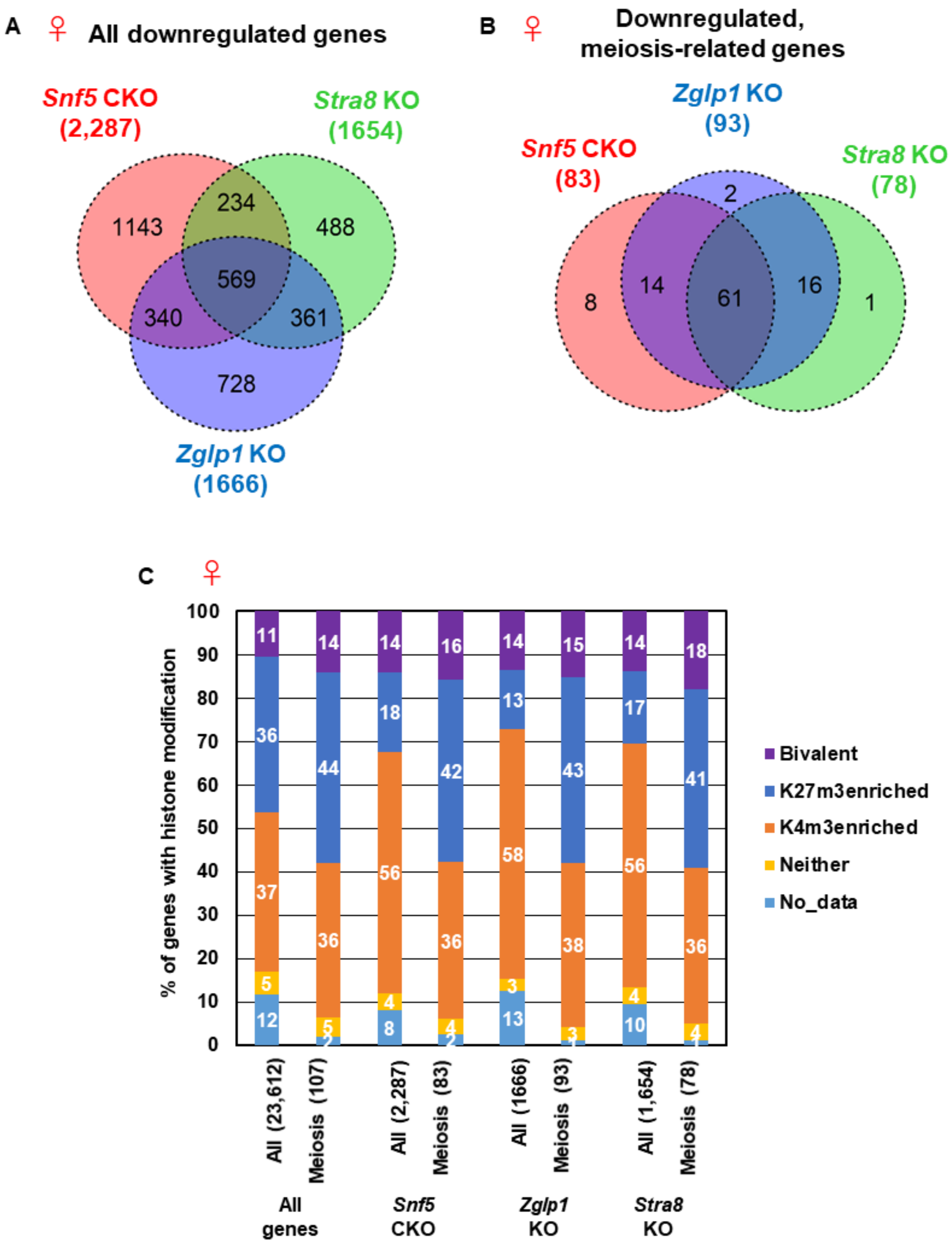

Figure S7. Target genes of the SWI/SNF complex, STRA8, and ZGLP1 in female germ cells.

(A, B) Venn diagrams showing all downregulated genes (A) and downregulated meiosis-related genes (B). Datasets from E15.5 *Snf5* CKO female germ cells and oocyte-like cells induced from ES cells lacking *Stra8* or *Zglp1* were used for the analyses, as described in the Supplementary Materials and Methods.

(C) The histone modifications in the promoters of the downregulated genes described in A and B. The histone modifications in PGC-like cells (PGCLCs) prior to sex-dependent differentiation are shown. See Supplementary Materials and Methods for further details.

Supplementary Tables

Table S1. Fertility of the Snf5 CKO females (A) and males (B).

A. Female CKO mice

Genotype	Mouse ID	Mating period (days)	No of delivery	No of pups
Ctrl	2067	78	2	7, 8
	2075	50	2	7, 8
	2063	62	0	0
	2124	36	1	10
	2140	36	1	9
	2209	70	1	6, 8
	2185	70	1	9
	2210	55	1	8
	2327	105	2	7, 7
CKO	2069	62	1	1
	2074	50	1	4
	2062	62	0	0
	2122	36	0	0
	2142	36	0	0
	2208	70	1	7
	2186	70	2	6, 4
	2324	55	0	0
	2326	105	2	6, 6

B. Male CKO mice

Genotype	Mouse ID	Mating period (days)	No of delivery	No of pups
Ctrl	2058	62	2	7, 6
	2180	70	3	6, 6, 7
	2292	55	3	5, 5, 7
	2291	55	1	6
	2293	71	2	4, 4
CKO	2059	62	1	2
	2182	70	1	9
	2290	55	2	9, 4
	2337	68	1	6

Table S2 Primers and PCR conditions used for genotyping.

(A) List of PCR Primers

Name	Sequences
Snf5 CE	5’-CACCATGCCCCCACCTCCCCTACA-3’
Snf5 CER	5’-CAGGAAAATGGATGCAACTAAGAT-3’
Snf5 Δ1	5’-CCAGTTAAAAGCACTGACTGCTT-3’
pGKpA/U1	5’-TAAGGGCCAGCTCATTCCTCC-3’
TNAP/EX6L	5’-CACGTCGATGGCCGCTCTA-3’
EGFP-Fw	5’-AGCAAGGGCGAGGAGCTGTT-3’
EGFP-Rv	5’-GTAGGTCAGGGTGGTCACGA-3’

(B) Primer pairs, PCR conditions and the sizes of amplified DNAs

Allele	Primer set	Annealing temperature (X)	Cycle (Y)	PCR product
Snf5 WT and fx	Snf5 CE	62℃	35	WT: 200 bp
	Snf5 CER			fx: 305 bp
Snf5 Δ	Snf5 CE	60℃	30	Δ： 300 bp
	Snf5 Δ1			
TNAP-Cre	pGKpA/U1	65℃	30	TNAP-Cre: 300 bp
	TNAP/EX6L			
Oct4-EGFP	EGFP-Fw	65℃	30	Oct4-EGFP: 200 bp
	EGFP-Rv			

(C) PCR protocol

PCR		
94℃	5 min	
94℃	10 sec	Y cycles
X℃	10 sec	
72℃	30 sec	
12℃	Infinity	

Table S3 The antibodies used for immunohistochemistry.

Antibodies	Host	Type	Source	Ref	Dilution
anti-MVH	Rabbit	IgG	Abcam	ab13840	1/500
anti-TRA98	Rat	IgG	Abcam	ab82527	1/500
anti-SYCP3	Mouse	IgG	Abcam	ab97672	1/500
anti-KI67	Mouse	IgG	BD Pharmingen	550609	1/500
anti-phospho-histone H3	Rabbit	IgG	Upstate Biotech.	06-570	1/500
anti-MILI	Rabbit	-	Dr. S Kuramochi-Miyagawa	[2]	1/400
anti-MIWI2	Mouse	-	Dr. S Kuramochi-Miyagawa	[2]	1/2000
anti-DNMT3A	Rabbit	-	Dr. I Suetake-	[3]	1/500
anti-DNMT3L	Rabbit	-	Dr. I Suetake-	[3]	1/500
anti-MAEL	Rabbit	-	Dr. A Bortvin	[4]	1/500
Anti-SNF5	Mouse	IgG	BD Biosciences	612111	1/50 or 1/100
anti-Rabbit-IgG, CF488-conjugated	Goat	IgG	Biotium	20019	1/1000
anti-Mouse-IgG, CF568-conjugated	Goat	IgG	Biotium	20100	1/1000
anti-Rat-IgG, CF568-conjugated	Goat	IgG	Biotium	20096	1/1000
anti-Mouse EnVision+ System-HRP Labelled Polymer	-	-	Dako	K4001	Following to the manufacturer's recommendation

Table S4 The antibodies used for immunostaining of the chromatin spreads.

Antibodies	Host	Type	Source	Ref	Dilution
anti-SYCP1	Rabbit	IgG	Abcam	ab15090	1/500
anti-SYCP3	Mouse	IgG	Abcam	ab97672	1/500
anti-SYCP3	Rabbit	IgG	Dr. S Chuma	[5]	1/500
anti-γH2AX	Mouse	IgG	Abcam	ab22551	1/500
anti-Rabbit-IgG, CF488-conjugated	Goat	IgG	Biotium	20019	1/1000
anti-Mouse-IgG, CF568-conjugated	Goat	IgG	Biotium	20100	1/1000

Table S5. The number and the purity of germ cells.

Assay	Age	Genotype	Sex	No of mice	No of cells	Purity (%)
RNA-Seq	E15.5	Control-1	Female	2	8568	99
RNA-Seq	E15.5	Control-2	Female	3	11568	99
RNA-Seq	E15.5	CKO-1	Female	2	9228	99
RNA-Seq	E15.5	CKO-2	Female	3	9960	100
RNA-Seq	E15.5	Control-1	Male	6	3756	95
RNA-Seq	E15.5	Control-2	Male	4	21084	97
RNA-Seq	E15.5	CKO-1	Male	1	3228	96
RNA-Seq	E15.5	CKO-1	Male	1	2616	95
RNA-Seq	E15.5	CKO-2	Male	1	5640	100
BS-Seq	E17.5	Control-1	Male	4	3480	97
BS-Seq	E17.5	Control-2	Male	11	4356	97
BS-Seq	E17.5	CKO-1	Male	3	2712	93
BS-Seq	E17.5	CKO-2	Male	1	1140	98
BS-Seq	E18.5	Control-1	Male	7	1339	97
BS-Seq	E18.5	Control-2	Male	2	802	92
BS-Seq	E18.5	CKO-1	Male	1	132	100
BS-Seq	E19.5	Control-1	Male	3	2064	96
BS-Seq	E19.5	CKO-1	Male	1	168	93

Table S6 Sequences of the primers used in BS-Seq analyses.

Targets	Sequences
IAP	5'-TTGTGTTTTAAGTGGTAAATAAATAATTTG-3'<br>5'-AAAACACCACAAACCAAAATCTTCTAC-3'
Type A LINE1	5'-TTATTTTGATAGTAGAGTT-3'<br>5'-CAAACCAAACTCCTAACAA-3'
Type Tf LINE1	5'-GTTAGAGAATTTGATAGTTTTTGGAATAGG-3'<br>5'-CCAAAACAAAACCTTTCTCAAACACTATAT-3'
H19-DMR	5'-GGGGGTAGGATATATGTATTTTTTAGG-3'<br>5'-AAAAAAACTCAATCAATTACAATCC-3'
IG-DMR	5'-TTTTATGGTTTATTGTATATAATGTTGT-3'<br>5'-CCCTCACTCCAAAAATTAAAAAAA-3'

The target gene sequences without adapters used for Nextera tagmentation are shown.

Supplementary Methods

Estimation of Cre-mediated recombination efficiencies in *Snf5* loci

Using the RNA-Seq data of the E15.5 germ cells, recombination efficiency was estimated using the number of reads corresponding to exon 1 of the *Snf* gene, which is deleted by Cre-mediated recombination.

$$0.5\mathrm{R}WT\ (1-x) = \mathrm{R}CKO \quad \text{(Equation 1)}$$

$$\frac{\mathrm{R}WT\ \{\mathrm{N1} + 0.5\mathrm{N2} + \mathrm{N3}\ (1-0.5x)\ \}}{\mathrm{N1} + \mathrm{N2} + \mathrm{N3}} = \mathrm{R}Control \quad \text{(Equation 2)}$$

where x is the recombination efficiency. $\mathrm{R}WT$, $\mathrm{R}Control$, and $\mathrm{R}CKO$ indicate the number of reads corresponding to exon 1 in wild-type, control, and *Snf5* CKO mice, respectively. As the samples from control mice constituted a mixture of germ cells from $Snf5^{fx/+}$ mice, $Snf5^{fx/\Delta}$ mice, and $Snf5^{fx/+}$ mice harboring the *TNAP-Cre* allele, N1, N2, and N3 indicate the number of $Snf5^{fx/+}$ mice, $Snf5^{fx/\Delta}$ mice, and $Snf5^{fx/+}$ mice harboring the *TNAP-Cre* allele, respectively.

By dividing Equation 1 by Equation 2, the recombination efficiency x can be calculated as follows:

$$\therefore x = \frac{\mathrm{R}CKO(\mathrm{N1} + 0.5\mathrm{N2} + \mathrm{N3}) - 0.5\ \mathrm{R}Control(\mathrm{N1} + \mathrm{N2} + \mathrm{N3})}{0.5\mathrm{R}CKO\mathrm{N3} - 0.5\ \mathrm{R}Control(\mathrm{N1} + \mathrm{N2} + \mathrm{N3})} \quad \text{(Equation 3)}$$

TUNEL assay

The terminal deoxynucleotidyl transferase dUTP nick end labeling (TUNEL) assay was carried out using an *In situ* Apoptosis Detection Kit (Takara, Shiga, Japan) and the nuclei were counterstained with 1 μg/ml DAPI.

Target genes of the SWI/SNF complex, STRA8, and ZGLP1 in female germ cells.

RNA-seq analysis was performed as described in the Materials and Methods section. GSE124262 was used to analyze *Zglp1* and *Stra8* KO oocyte-like cells [6]. Downregulated genes were selected as those with $\log_2$(FPKM) ≤ -1 and $P < 0.05$. A published list of meiosis-related genes was used [7]. The R packages VennDiagram and extrafont were used to draw the Venn diagrams. Previously published data were used to investigate histone modifications [6]. MS Excel (Microsoft, Redmond, WA, USA) was used for the calculations and generation of the bar graph. VLOOKUP was used to search for the histone modifications of each gene.

Supplementary References

1. Miyauchi, H. *et al.* Bone morphogenetic protein and retinoic acid synergistically specify female germ-cell fate in mice. *EMBO J.* **36**, 3100–3119 (2017).

2. Shiromoto, Y. *et al.* GPAT2 is required for piRNA biogenesis, transposon silencing, and maintenance of spermatogonia in mice. *Biol. Reprod.* **101**, 248–256 (2019).

3. Sakai, Y., Suetake, I., Shinozaki, F., Yamashina, S. & Tajima, S. Co-expression of de novo DNA methyltransferases Dnmt3a2 and Dnmt3L in gonocytes of mouse embryos. *Gene Expr. Patterns* **5**, 231–237 (2004).

4. Soper, S. F. C. *et al.* Mouse Maelstrom, a Component of Nuage, Is Essential for Spermatogenesis and Transposon Repression in Meiosis. *Dev. Cell* **15**, 285–297 (2008).

5. Chuma, S. & Nakatsuji, N. Autonomous transition into meiosis of mouse fetal germ cells in vitro and its inhibition by gp130-mediated signaling. *Dev. Biol.* **229**, 468–479 (2001).

6. Nagaoka, S. I. *et al.* ZGLP1 is a determinant for the oogenic fate in mice. *Science* **4115**, eaaw4115 (2020).

7. Soh, Y. Q. S. *et al.* A Gene Regulatory Program for Meiotic Prophase in the Fetal Ovary. *PLoS Genet.* **11**, 1–24 (2015).
